# Supplementary material for: Vegetation and vertebrate abundance as drivers of bioturbation patterns along a climate gradient
Source: PLoS One. 2022 Mar 4;17(3):e0264408. doi: 10.1371/journal.pone.0264408 (PMC8896722; doi:10.1371/journal.pone.0264408)
Supplement: S1 Appendix — (DOCX) [file pone.0264408.s001.docx]

**I Raw data of bioturbation parameters**

For comparison with published studies, in the following we report the mean, standard deviation and range of the raw data. For the same reason, we converted our results on excavated soil volume [100 cm³ * (100 m²)^-1^] to [m³ ha^-1^]. We did not include the annual excavated soil volume since the observed differences in bioturbation quantity were seasonal and burrows from previous seasons were not sampled.

**Table S1: Raw data of the bioturbation parameters hole density and excavated soil volume.** Mean, SD = standard deviation and range of raw data of hole density and calculated excavated soil volume. NP = National Park. Data from the field campaign from September to November were used.

| Parameter | Site | Animal group | Mean | SD | Range |
| --- | --- | --- | --- | --- | --- |
| Hole density [no (100 m²)^-1^] | NP Pan de Azúcar | vertebrate | 2.8 | 2.9 | 0 - 10 |
|  |  | invertebrate | 14 | 7.8 | 3 - 28 |
|  | Santa Gracia | vertebrate | 9.1 | 9.7 | 1 - 37 |
|  |  | invertebrate | 7.6 | 6.5 | 0 - 26 |
|  | NP La Campana | vertebrate | 5.6 | 8.7 | 0 - 33 |
|  |  | invertebrate | 6.8 | 5.1 | 0 - 16 |
|  | NP Nahuelbuta | vertebrate | 2.2 | 2.1 | 0 - 6 |
|  |  | invertebrate | 2.8 | 3.1 | 0 - 10 |
| Excavated soil volume [m³ ha^-1^] | NP Pan de Azúcar | vertebrate | 0.01 | 0.021 | 0 - 0.089 |
|  |  | invertebrate | 0.000 31 | 0.0002 | 0.00005 - 0.00081 |
|  | Santa Gracia | vertebrate | 0.034 | 0.063 | 0.00017 - 0.24 |
|  |  | invertebrate | 0.00056 | 0.00064 | 0 - 0.0027 |
|  | NP La Campana | vertebrate | 0.06 | 0.18 | 0 - 0.82 |
|  |  | invertebrate | 0.00019 | 0.00016 | 0 - 0.00065 |
|  | NP Nahuelbuta | vertebrate | 0.012 | 0.02 | 0 - 0.068 |
|  |  | invertebrate | 0.00015 | 0.00022 | 0 - 0.00096 |
