# Supplementary material for: Vegetation and vertebrate abundance as drivers of bioturbation patterns along a climate gradient
Source: PLoS One. 2022 Mar 4;17(3):e0264408. doi: 10.1371/journal.pone.0264408 (PMC8896722; doi:10.1371/journal.pone.0264408)
Supplement: S2 Appendix — (DOCX) [file pone.0264408.s002.docx]

**II Relationships between measured hole variables**

We performed regressions between hole depth and diameter as well as between excavated soil volume and hole density for only one season (Chilean spring in September, October, November) to compare seasonal patterns and overall patterns. We chose this season because it consisted of more plots (20) than established in the preceding autumn (12).


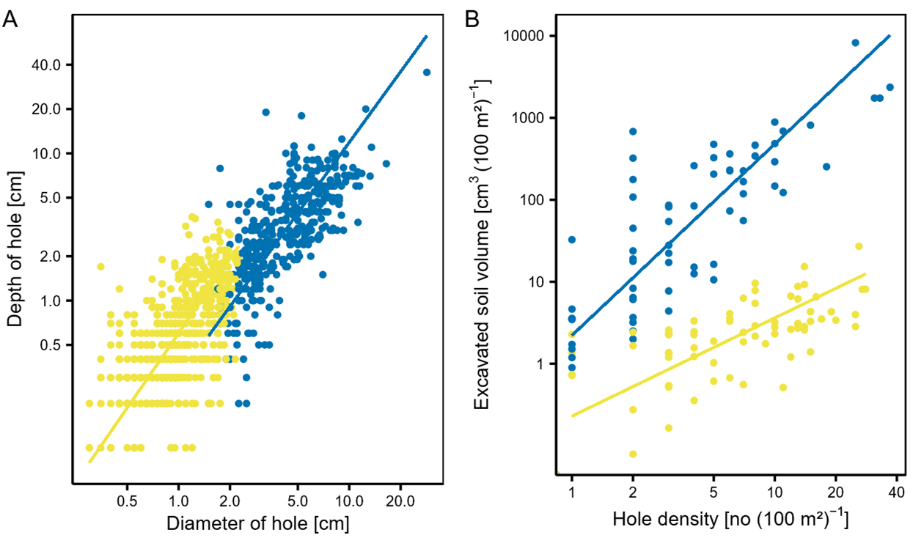


**S1 Fig. Relationships between burrow characteristics and bioturbation parameters created by burrowing invertebrates (yellow) and vertebrates (blue).** (A) Relationship between the depth and mean diameter of the holes, (B) relationship between the excavated soil volume and hole density. The regression lines are from the reduced major axis analysis. Note that both axes in (A) and (B) were log_10_-scaled. Data from the field campaign from September to November and all sites were used.

**S2 Table. Ordinary least squares (OLS) and reduced major axis (RMA) regression analyses of the relationships between the depth and mean diameter of the holes and between the excavated soil volume and hole density for vertebrates and invertebrates (all variables log_10_-transformed).**

A slope of one represents an isometric relationship. The same was applied in the OLS analysis with plot number as a random factor. Depicted are statistical method, correlation coefficient, slope, standard error (SE) and p-value (p) of the offset. Significant effects are labelled with asterisks: *:<0.1, **:<0.01, ***:<0.001. Data from the field campaign from September to November and from all sites were used. Further information on the statistical analysis is provided in the Methods section.

|  |  | invertebrate | | | | vertebrate | | | |
| --- | --- | --- | --- | --- | --- | --- | --- | --- | --- |
| Relation | method | r | slope | SE | p | r | slope | SE | p |
| Depth and diameter | OLS  (mixed model) | 0.40 | 1.04 | 0.053 | <0.001*** | 0.49 | 1.12 | 0.057 | <0.001*** |
|  | OLS | 0.61 | 1.03 | 0.053 | 0.62 | 0.7 | 1.12 | 0.057 | 0.0332* |
|  | RMA | 0.57 | 1.68 | 0.053 | <0.001*** | 0.56 | 1.59 | 0.057 | <0.001*** |
| Excavated soil volume and hole density | OLS  (mixed model) | 0.35 | 0.712 | 0.12 | 0.017* | 0.66 | 1.91 | 0.17 | <0.001*** |
|  | OLS | 0.59 | 0.712 | 0.12 | 0.017* | 0.82 | 1.91 | 0.17 | <0.001*** |
|  | RMA | 0.22 | 1.2 | 0.12 | <0.001*** | 0.86 | 2.34 | 0.17 | <0.001*** |
