# Supplementary material for: Vegetation and vertebrate abundance as drivers of bioturbation patterns along a climate gradient
Source: PLoS One. 2022 Mar 4;17(3):e0264408. doi: 10.1371/journal.pone.0264408 (PMC8896722; doi:10.1371/journal.pone.0264408)
Supplement: S3 Appendix — (DOCX) [file pone.0264408.s003.docx]

**III Results of GLMMs and the relationships between bioturbation parameters and vegetation cover**

We performed GLMMs followed by Chi-squared tests to compare the different models with hole density and excavated soil volume as the predictor variables. For clarity, the relationship between hole density and excavated soil volume against vegetation cover was then plotted.

**S3 Table. Results of the GLMM for hole density at each site** **(Pan de Azúcar, Santa Gracia, La Campana, Nahuelbuta) for both seasons (autumn: March-April/ spring: September-November) and both animal groups (invertebrates/ vertebrates).** Depicted are the fixed effects for the predictors, the estimate, the standard error SE and the z- and p-value. Data from both field campaigns were used. Significant effects are labelled with asterisks: *:<0.1, **:<0.01, ***:<0.001.

| Fixed effects | Estimate | SE | z | p |
| --- | --- | --- | --- | --- |
| (Intercept) | 2.80 | 0.117 | 23.9 | <0.001 *** |
| Site Santa Gracia | -0.454 | 0.194 | -2.34 | 0.019 * |
| Site La Campana | -1.32 | 0.238 | -5.53 | <0.001 *** |
| Site Nahuelbuta | 1.88 | 0.801 | 2.34 | 0.019 * |
| Hillslope | 0.0245 | 0.00404 | 6.05 | <0.001 *** |
| Animal group vertebrate | -1.56 | 0.0993 | -15.7 | <0.001 *** |
| Season September-November | -0.381 | 0.0795 | -4.8 | <0.001 *** |
| Hillside elevation | -0.00495 | 0.0421 | -0.118 | 0.91 |
| Vegetation cover | -0.0702 | 0.0101 | -6.92 | <0.001 *** |
| Site Santa Gracia: Taxon vertebrate | 0.726 | 0.141 | 5.14 | <0.001 *** |
| Site La Campana: Taxon vertebrate | -0.969 | 0.259 | -3.74 | <0.001 *** |
| Site Nahuelbuta: Taxon vertebrate | -2.81 | 0.393 | -7.15 | <0.001 *** |
| Site Santa Gracia: Season September-November | -0.0673 | 0.112 | -0.602 | 0.55 |
| Site La Campana: Season September-November | 0.643 | 0.137 | 4.70 | <0.001 *** |
| Site Nahuelbuta: Season September-November | -0.0911 | 0.175 | -0.521 | 0.6 |
| Site Santa Gracia: Hillslope | 0.026 | 0.0073 | 3.56 | <0.001 *** |
| Site La Campana: Hillslope | 0.00371 | 0.0069 | 0.538 | 0.59 |
| Site Nahuelbuta: Hillslope | 0.0445 | 0.0151 | 2.94 | 0.0033 ** |
| Site Santa Gracia: Hillside elevation | -0.099 | 0.0586 | -1.69 | 0.09 |
| Site La Campana: Hillside elevation | 0.158 | 0.076 | 2.07 | 0.038 * |
| Site Nahuelbuta: Hillside elevation | 0.719 | 0.138 | 5.21 | <0.001 *** |
| Animal group vertebrate: Vegetation cover | 0.045 | 0.00389 | 11.6 | <0.001 *** |
| Site Santa Gracia: Vegetation cover | 0.0292 | 0.011 | 2.66 | 0.0078 ** |
| Site La Campana: Vegetation cover | 0.0583 | 0.0104 | 5.63 | <0.001 *** |
| Site Nahuelbuta: Vegetation cover | 0.0205 | 0.0126 | 1.62 | 0.1 |

**S4 Table. Results of the GLMM for the excavated soil volume (log_10_-transformed) at each site (Pan de Azúcar, Santa Gracia, La Campana, Nahuelbuta) for both seasons (autumn: March-April/ spring: September-November) and both animal groups (invertebrates/ vertebrates).** Depicted are the fixed effects for the predictors, the estimate, the standard error (SE) and the t- and p-value. Data from both field campaigns were used. Significant effects are labelled with asterisks: *:<0.1, **:<0.01, ***:<0.001.

| Fixed effects | Estimate | SE | t | p |
| --- | --- | --- | --- | --- |
| (Intercept) | -0.322 | 0.205 | -1.57 | 0.12 |
| Animal group vertebrate | 1.08 | 0.231 | 4.66 | <0.001 *** |
| Season September-November | -0.233 | 0.0607 | -3.84 | <0.001 *** |
| Vegetation cover | 0.003 | 0.0134 | 0.224 | 0.82 |
| Hillside elevation | -0.107 | 0.0534 | -1.99 | 0.048 * |
| Site Santa Gracia | 0.374 | 0.198 | 1.89 | 0.06 |
| Site La Campana | -0.274 | 0.204 | -1.34 | 0.18 |
| Site Nahuelbuta | -2.02 | 1.06 | -1.9 | 0.059 |
| Animal group invertebrate: Hole density | 0.988 | 0.134 | 7.36 | <0.001*** |
| Animal group vertebrate: Hole density | 1.82 | 0.107 | 17.0 | <0.001*** |
| Animal group vertebrate: Vegetation cover | -0.0112 | 0.0187 | -0.602 | 0.55 |
| Animal group vertebrate: Site Santa Gracia | -0.598 | 0.281 | -2.13 | 0.034 * |
| Animal group vertebrate: Site La Campana | 1.1 | 0.339 | 3.25 | 0.0014 ** |
| Animal group vertebrate: Site Nahuelbuta | 4.19 | 1.46 | 2.87 | 0.0045 ** |
| Hillside elevation: Site Santa Gracia | 0.169 | 0.0756 | 2.23 | 0.027 * |
| Hillside elevation: Site La Campana | 0.198 | 0.0789 | 2.51 | 0.013 * |
| Hillside elevation: Site Nahuelbuta | 0.172 | 0.107 | 1.61 | 0.11 |
| Vegetation cover: Site Santa Gracia | -0.00509 | 0.0147 | -0.347 | 0.73 |
| Vegetation cover: Site La Campana | 0.00121 | 0.0135 | 0.089 | 0.93 |
| Vegetation cover: Site Nahuelbuta | 0.0208 | 0.0174 | 1.19 | 0.23 |
| Animal group vertebrate: Vegetation cover: Site Santa Gracia | 0.0179 | 0.0208 | 0.862 | 0.39 |
| Animal group vertebrate: Vegetation cover: Site La Campana | -0.000534 | 0.0193 | -0.028 | 0.98 |
| Animal group vertebrate: Vegetation cover: Site Nahuelbuta | -0.0327 | 0.024 | -1.36 | 0.18 |

**S5 Table. Chi-squared tests to compare different models of fixed predictors in GLMMs for the response variable hole density.** Depicted are the different combinations of the GLMM (Null: tests the null hypothesis against 1 without any fixed predictor), npar: model parameters; AIC, BIC, logLik: log-likelihood for the model; deviance, Chisq: Chi-square-statistic, Df: degrees of freedom and p: p-value. S=site, Sl=hillslope, E=hillside elevation, V=vegetation cover, Se=Season, A=animal group, Pn=plot number. Significant effects are labelled with asterisks: *:<0.1, **:<0.01, ***:<0.001.

| GLMM | npar | AIC | BIC | logLik | deviance | Chisq | Df | p |
| --- | --- | --- | --- | --- | --- | --- | --- | --- |
| Null | 2 | 2267.1 | 2274.2 | -1131.57 | 2263.1 |  |  |  |
| S:Se + S:V:A + (1\|Pn) | 17 | 2052.1 | 2112.4 | -1009.04 | 2018.1 | 144 | 4 | <0.001 *** |
| S:Se + S:E + (1\|Pn) | 13 | 2185.6 | 2231.7 | -1079.8 | 2159.6 | 104 | 11 | <0.001 *** |
| S:Se + S:Sl + (1\|Pn) | 13 | 2188.3 | 2234.4 | -1081.16 | 2162.3 | 0 | 0 |  |
| S:Se + S:V:A + S:E + (1\|Pn) | 21 | 2046.4 | 2120.9 | -1002.22 | 2004.4 | 36.9 | 3 | <0.001 *** |
| S:Se + S:V:A + S:Sl + (1\|Pn) | 21 | 2050 | 2124.4 | -1004 | 2008 | 0 | 0 |  |
| S:V:A + S:E + S:Sl + (1\|Pn) | 18 | 2077.3 | 2141.2 | -1020.67 | 2041.3 | 98.09 | 1 | <0.001 *** |
| S:Se + S:E + S:Sl + (1\|Pn) | 17 | 2173.4 | 2233.7 | -1069.71 | 2139.4 | 0 | 0 |  |
| S:Se + S:V:A + S:E + S:Sl + (1\|Pn) | 25 | 2030.7 | 2119.3 | -990.34 | 1980.7 | 27.3 | 4 | <0.001 *** |

**S6 Table. Chi-squared tests to compare different models of fixed predictors in GLMMs for the response variable excavated soil volume.** Depicted are the different combinations of the GLMM (Null: tests the null hypothesis against 1 without any fixed predictor), npar: model parameters; AIC, BIC, logLik: log-likelihood for the model; deviance, Chisq: Chi-square-statistic, Df: degrees of freedom and p: p-value. Hd=hole density, S=site, Sl=hillslope, E=hillside elevation, V=vegetation cover, Se=Season, A=animal group. Significant effects are labelled with asterisks: *:<0.1, **:<0.01, ***:<0.001.

| GLMM | npar | AIC | BIC | logLik | deviance | Chisq | Df | p |
| --- | --- | --- | --- | --- | --- | --- | --- | --- |
| Null | 3 | 612.19 | 622.23 | -303.09 | 606.19 |  |  |  |
| Se + S:V:A | 12 | 472.1 | 512.27 | -224.05 | 448.1 | 0 | 2 | 1 |
| Se + A:Hd | 6 | 330.71 | 350.79 | -159.35 | 318.71 | 287 | 3 | <0.001 *** |
| Se + S:E | 8 | 610.36 | 637.14 | -297.18 | 594.36 | 0 | 2 | 1 |
| Se + S:V:A + A:Hd | 14 | 296.67 | 343.53 | -134.34 | 268.67 | 179 | 2 | <0.001 *** |
| S:V:A + A:Hd + S:E | 17 | 309.87 | 366.77 | -137.93 | 275.87 | 171 | 1 | <0.001 *** |
| Se + A:Hd + S:E | 10 | 330.55 | 364.02 | -155.28 | 310.55 | 284 | 2 | <0.001 *** |
| Se + S:V:A + S:E | 16 | 478.63 | 532.19 | -223.32 | 446.63 | 0 | 2 | 1 |
| Se + S:V:A + A:Hd + S:E | 18 | 296.67 | 356.92 | -130.34 | 260.67 | 15.2 | 1 | <0.001 *** |


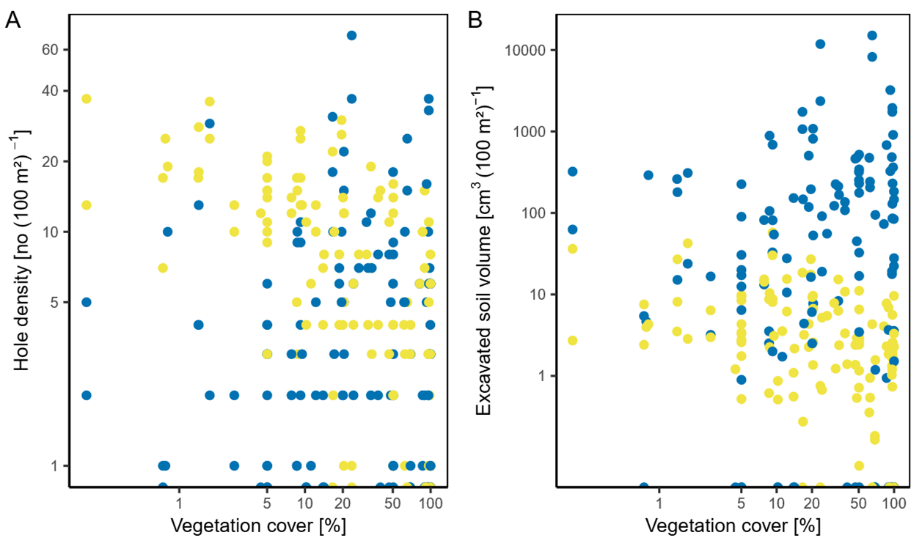


**S2 Fig. Relationships between bioturbation parameters and vegetation cover.** (A) Relationship between hole density (log_10_-transformed) and vegetation cover (log_10_-transformed), (B) relationship between excavated soil volume (log_10_-transformed) and vegetation cover of invertebrates (yellow) and vertebrates (blue). Note that both axes in (a) and (B) were log_10_-scaled. Data from both campaigns were used.
