# Supplementary material for: Vegetation and vertebrate abundance as drivers of bioturbation patterns along a climate gradient
Source: PLoS One. 2022 Mar 4;17(3):e0264408. doi: 10.1371/journal.pone.0264408 (PMC8896722; doi:10.1371/journal.pone.0264408)
Supplement: S4 Appendix — (DOCX) [file pone.0264408.s004.docx]

**IV Raw data for calculations**

**S7 Table. Raw data of hole density and burrow parameters and data for GLMM calculations.**

| Field campaign | Research site | Plot number | Diameter vertical [cm] | Diameter horizontal [cm] | Depth [cm] |
| --- | --- | --- | --- | --- | --- |
| 1st | Pan de Azúcar | 1 | 4.3 | 2.4 | 5.5 |
| 1st | Pan de Azúcar | 1 | 6.7 | 6.8 | 4 |
| 1st | Pan de Azúcar | 1 | 3.4 | 3.5 | 4 |
| 1st | Pan de Azúcar | 1 | 3.7 | 2.9 | 1.5 |
| 1st | Pan de Azúcar | 1 | 0.9 | 0.9 | 1.5 |
| 1st | Pan de Azúcar | 1 | 1 | 1 | 1.7 |
| 1st | Pan de Azúcar | 1 | 0.9 | 1 | 1.9 |
| 1st | Pan de Azúcar | 1 | 3.2 | 4 | 2 |
| 1st | Pan de Azúcar | 1 | 1.1 | 1.5 | 1.8 |
| 1st | Pan de Azúcar | 1 | 3.1 | 1.8 | 2.5 |
| 1st | Pan de Azúcar | 2 | 0.9 | 1 | 3.9 |
| 1st | Pan de Azúcar | 2 | 1 | 2 | 3.1 |
| 1st | Pan de Azúcar | 2 | 1 | 1 | 2.5 |
| 1st | Pan de Azúcar | 2 | 1 | 1 | 1.8 |
| 1st | Pan de Azúcar | 2 | 0.5 | 0.5 | 2.2 |
| 1st | Pan de Azúcar | 2 | 3 | 3 | 2.4 |
| 1st | Pan de Azúcar | 2 | 1 | 5 | 2 |
| 1st | Pan de Azúcar | 2 | 2 | 1.5 | 1.9 |
| 1st | Pan de Azúcar | 2 | 1 | 1 | 1.2 |
| 1st | Pan de Azúcar | 2 | 3 | 5 | 3 |
| 1st | Pan de Azúcar | 2 | 1.5 | 2 | 3.5 |
| 1st | Pan de Azúcar | 2 | 2.5 | 3 | 1.9 |
| 1st | Pan de Azúcar | 2 | 1 | 1 | 2.4 |
| 1st | Pan de Azúcar | 2 | 2.5 | 4 | 3.8 |
| 1st | Pan de Azúcar | 2 | 2 | 1.5 | 2.4 |
| 1st | Pan de Azúcar | 2 | 2.5 | 2 | 2.8 |
| 1st | Pan de Azúcar | 2 | 1 | 2 | 2.9 |
| 1st | Pan de Azúcar | 2 | 3 | 2.5 | 3.2 |
| 1st | Pan de Azúcar | 2 | 3 | 3.5 | 3 |
| 1st | Pan de Azúcar | 2 | 0.8 | 0.9 | 1.3 |
| 1st | Pan de Azúcar | 2 | 1 | 0.8 | 1 |
| 1st | Pan de Azúcar | 2 | 3 | 2 | 2.9 |
| 1st | Pan de Azúcar | 2 | 1.5 | 2 | 1.2 |
| 1st | Pan de Azúcar | 2 | 0.6 | 1 | 2.2 |
| 1st | Pan de Azúcar | 2 | 3.5 | 4 | 3.5 |
| 1st | Pan de Azúcar | 2 | 1.5 | 3 | 1 |
| 1st | Pan de Azúcar | 2 | 1 | 1 | 2.1 |
| 1st | Pan de Azúcar | 2 | 0.5 | 0.5 | 1.2 |
| 1st | Pan de Azúcar | 2 | 1 | 1 | 3.2 |
| 1st | Pan de Azúcar | 2 | 2 | 1.5 | 3.4 |
| 1st | Pan de Azúcar | 2 | 2 | 2.5 | 4.5 |
| 1st | Pan de Azúcar | 2 | 3 | 3 | 4 |
| 1st | Pan de Azúcar | 2 | 2 | 2 | 4.2 |
| 1st | Pan de Azúcar | 2 | 1.5 | 2.5 | 3.5 |
| 1st | Pan de Azúcar | 2 | 0.6 | 0.6 | 1 |
| 1st | Pan de Azúcar | 2 | 0.7 | 0.6 | 3.3 |
| 1st | Pan de Azúcar | 2 | 2.2 | 2.4 | 2.9 |
| 1st | Pan de Azúcar | 2 | 0.8 | 0.9 | 4 |
| 1st | Pan de Azúcar | 2 | 0.6 | 0.4 | 1.3 |
| 1st | Pan de Azúcar | 2 | 1 | 1.2 | 1.7 |
| 1st | Pan de Azúcar | 2 | 2.5 | 3 | 3.5 |
| 1st | Pan de Azúcar | 2 | 2 | 4 | 1 |
| 1st | Pan de Azúcar | 2 | 5 | 5 | 4 |
| 1st | Pan de Azúcar | 2 | 4 | 4.5 | 2.5 |
| 1st | Pan de Azúcar | 2 | 1.5 | 2 | 2.9 |
| 1st | Pan de Azúcar | 2 | 4.5 | 4.5 | 5.5 |
| 1st | Pan de Azúcar | 2 | 3 | 4.5 | 4 |
| 1st | Pan de Azúcar | 2 | 2 | 1.7 | 2.8 |
| 1st | Pan de Azúcar | 2 | 1.2 | 1 | 2.3 |
| 1st | Pan de Azúcar | 2 | 2.5 | 3.5 | 0.5 |
| 1st | Pan de Azúcar | 2 | 2.5 | 4 | 2 |
| 1st | Pan de Azúcar | 2 | 3 | 2.5 | 1.5 |
| 1st | Pan de Azúcar | 2 | 2.5 | 3.5 | 2 |
| 1st | Pan de Azúcar | 2 | 3 | 2.5 | 1 |
| 1st | Pan de Azúcar | 2 | 2 | 2.5 | 0.5 |
| 1st | Pan de Azúcar | 2 | 2.5 | 3 | 3 |
| 1st | Pan de Azúcar | 2 | 1.5 | 1.7 | 4 |
| 1st | Pan de Azúcar | 2 | 1 | 1 | 3 |
| 1st | Pan de Azúcar | 2 | 1 | 1.2 | 3.5 |
| 1st | Pan de Azúcar | 2 | 1.5 | 1 | 3.2 |
| 1st | Pan de Azúcar | 2 | 1.2 | 1 | 2.5 |
| 1st | Pan de Azúcar | 2 | 5 | 8.5 | 7 |
| 1st | Pan de Azúcar | 2 | 3.5 | 4 | 5 |
| 1st | Pan de Azúcar | 2 | 0.9 | 0.8 | 1.6 |
| 1st | Pan de Azúcar | 2 | 1 | 2 | 1.3 |
| 1st | Pan de Azúcar | 3 | 1 | 2 | 2 |
| 1st | Pan de Azúcar | 3 | 1.2 | 4 | 1.8 |
| 1st | Pan de Azúcar | 3 | 1.1 | 1.5 | 1.8 |
| 1st | Pan de Azúcar | 3 | 2.8 | 3.5 | 3 |
| 1st | Pan de Azúcar | 3 | 1.2 | 2 | 4.5 |
| 1st | Pan de Azúcar | 3 | 3 | 3.5 | 3 |
| 1st | Pan de Azúcar | 3 | 2 | 2.5 | 4.8 |
| 1st | Pan de Azúcar | 3 | 0.5 | 0.5 | 2.9 |
| 1st | Pan de Azúcar | 3 | 0.3 | 0.8 | 3 |
| 1st | Pan de Azúcar | 3 | 2.4 | 3.2 | 3.8 |
| 1st | Pan de Azúcar | 3 | 0.5 | 0.6 | 2.9 |
| 1st | Pan de Azúcar | 3 | 0.5 | 0.5 | 2 |
| 1st | Pan de Azúcar | 3 | 1 | 0.6 | 3.5 |
| 1st | Pan de Azúcar | 3 | 0.5 | 0.4 | 0.9 |
| 1st | Pan de Azúcar | 3 | 0.6 | 1 | 1.4 |
| 1st | Pan de Azúcar | 3 | 2 | 3 | 4.5 |
| 1st | Pan de Azúcar | 3 | 0.6 | 1 | 2.4 |
| 1st | Pan de Azúcar | 3 | 0.6 | 0.6 | 2.2 |
| 1st | Pan de Azúcar | 3 | 0.5 | 0.7 | 1.9 |
| 1st | Pan de Azúcar | 3 | 1.5 | 1 | 1.5 |
| 1st | Pan de Azúcar | 3 | 3 | 5 | 7.5 |
| 1st | Pan de Azúcar | 3 | 4.5 | 5.5 | 4 |
| 1st | Pan de Azúcar | 3 | 2.5 | 5 | 2 |
| 1st | Pan de Azúcar | 3 | 1.5 | 1.5 | 4 |
| 1st | Pan de Azúcar | 4 | 3.2 | 2.7 | 4.4 |
| 1st | Pan de Azúcar | 4 | 4.5 | 4.4 | 7 |
| 1st | Pan de Azúcar | 4 | 2.3 | 3.4 | 3.2 |
| 1st | Pan de Azúcar | 4 | 1.5 | 1.5 | 3.5 |
| 1st | Pan de Azúcar | 4 | 0.6 | 0.8 | 1.4 |
| 1st | Pan de Azúcar | 4 | 0.5 | 0.4 | 3 |
| 1st | Pan de Azúcar | 4 | 0.8 | 0.8 | 2.3 |
| 1st | Pan de Azúcar | 4 | 1 | 1.5 | 3.2 |
| 1st | Pan de Azúcar | 4 | 1 | 0.8 | 1.5 |
| 1st | Pan de Azúcar | 4 | 0.6 | 1 | 3 |
| 1st | Pan de Azúcar | 4 | 1 | 0.7 | 3.2 |
| 1st | Pan de Azúcar | 4 | 1.5 | 2 | 4 |
| 1st | Pan de Azúcar | 4 | 2 | 1.5 | 2 |
| 1st | Pan de Azúcar | 4 | 1 | 1.5 | 4 |
| 1st | Pan de Azúcar | 4 | 0.8 | 1 | 2.3 |
| 1st | Pan de Azúcar | 4 | 1.5 | 1.5 | 3.1 |
| 1st | Pan de Azúcar | 4 | 0.5 | 0.7 | 2.6 |
| 1st | Pan de Azúcar | 4 | 0.4 | 0.4 | 2.4 |
| 1st | Pan de Azúcar | 4 | 0.8 | 0.6 | 2.3 |
| 1st | Pan de Azúcar | 4 | 1 | 1 | 3 |
| 1st | Pan de Azúcar | 4 | 1 | 1.5 | 2.6 |
| 1st | Pan de Azúcar | 4 | 1 | 0.7 | 1.4 |
| 1st | Pan de Azúcar | 4 | 1 | 0.8 | 2.3 |
| 1st | Pan de Azúcar | 4 | 0.6 | 1 | 3 |
| 1st | Pan de Azúcar | 4 | 0.4 | 0.5 | 2.3 |
| 1st | Pan de Azúcar | 4 | 0.4 | 0.5 | 2.3 |
| 1st | Pan de Azúcar | 4 | 1.5 | 1.5 | 3.6 |
| 1st | Pan de Azúcar | 4 | 1.5 | 2.5 | 3 |
| 1st | Pan de Azúcar | 4 | 1.5 | 1.5 | 6.1 |
| 1st | Pan de Azúcar | 4 | 0.4 | 0.6 | 2.1 |
| 1st | Pan de Azúcar | 4 | 1 | 0.8 | 2.2 |
| 1st | Pan de Azúcar | 4 | 0.5 | 0.5 | 1.8 |
| 1st | Pan de Azúcar | 4 | 1 | 1 | 3.5 |
| 1st | Pan de Azúcar | 4 | 1.3 | 1.5 | 2.5 |
| 1st | Pan de Azúcar | 4 | 1 | 1 | 1.9 |
| 1st | Pan de Azúcar | 4 | 0.5 | 0.5 | 3 |
| 1st | Pan de Azúcar | 4 | 4 | 3 | 2 |
| 1st | Pan de Azúcar | 4 | 2 | 1.8 | 6 |
| 1st | Pan de Azúcar | 4 | 1.2 | 1 | 2.3 |
| 1st | Pan de Azúcar | 4 | 1 | 1.2 | 6.4 |
| 1st | Pan de Azúcar | 4 | 0.6 | 0.6 | 2 |
| 1st | Pan de Azúcar | 4 | 0.5 | 0.5 | 1.3 |
| 1st | Pan de Azúcar | 5 | 0.4 | 0.4 | 2.5 |
| 1st | Pan de Azúcar | 5 | 0.4 | 1 | 1.6 |
| 1st | Pan de Azúcar | 5 | 1 | 1 | 4 |
| 1st | Pan de Azúcar | 5 | 0.5 | 0.5 | 4.9 |
| 1st | Pan de Azúcar | 5 | 1 | 1 | 3 |
| 1st | Pan de Azúcar | 5 | 1 | 0.7 | 2.9 |
| 1st | Pan de Azúcar | 5 | 0.5 | 0.4 | 0.4 |
| 1st | Pan de Azúcar | 5 | 0.5 | 0.5 | 2.2 |
| 1st | Pan de Azúcar | 5 | 3 | 1.5 | 4.1 |
| 1st | Pan de Azúcar | 5 | 1.3 | 1.5 | 2.4 |
| 1st | Pan de Azúcar | 5 | 1 | 0.1 | 4 |
| 1st | Pan de Azúcar | 5 | 0.9 | 1 | 0.8 |
| 1st | Pan de Azúcar | 5 | 0.8 | 0.7 | 1 |
| 1st | Pan de Azúcar | 5 | 1 | 1 | 3 |
| 1st | Pan de Azúcar | 5 | 1 | 1 | 1.9 |
| 1st | Pan de Azúcar | 5 | 1 | 1 | 3 |
| 1st | Pan de Azúcar | 5 | 0.5 | 0.6 | 2.2 |
| 1st | Pan de Azúcar | 5 | 0.8 | 0.8 | 1.4 |
| 1st | Pan de Azúcar | 6 | 1 | 1 | 2.3 |
| 1st | Pan de Azúcar | 6 | 1 | 0.8 | 3.1 |
| 1st | Pan de Azúcar | 6 | 1 | 0.8 | 3.1 |
| 1st | Pan de Azúcar | 6 | 0.8 | 1.2 | 2.6 |
| 1st | Pan de Azúcar | 6 | 0.7 | 0.8 | 1.5 |
| 1st | Pan de Azúcar | 6 | 1 | 1 | 1.5 |
| 1st | Pan de Azúcar | 6 | 2 | 2.5 | 1.9 |
| 1st | Pan de Azúcar | 6 | 0.4 | 0.4 | 1.7 |
| 1st | Pan de Azúcar | 6 | 0.7 | 0.5 | 1.1 |
| 1st | Pan de Azúcar | 6 | 0.7 | 1 | 1.2 |
| 1st | Pan de Azúcar | 6 | 1 | 1.5 | 1.8 |
| 1st | Pan de Azúcar | 6 | 0.9 | 1 | 1.9 |
| 1st | Pan de Azúcar | 6 | 0.9 | 1 | 1.9 |
| 1st | Pan de Azúcar | 6 | 1 | 1.2 | 4 |
| 1st | Pan de Azúcar | 6 | 0.5 | 0.6 | 1 |
| 1st | Pan de Azúcar | 6 | 0.6 | 1 | 1.3 |
| 1st | Pan de Azúcar | 6 | 1.2 | 1.3 | 2.8 |
| 1st | Pan de Azúcar | 6 | 1 | 0.9 | 3.2 |
| 1st | Pan de Azúcar | 7 | 0.8 | 0.7 | 1.5 |
| 1st | Pan de Azúcar | 7 | 1.2 | 1 | 3.2 |
| 1st | Pan de Azúcar | 7 | 0.7 | 0.8 | 1.8 |
| 1st | Pan de Azúcar | 7 | 0.8 | 0.8 | 3.3 |
| 1st | Pan de Azúcar | 7 | 1 | 0.9 | 2.7 |
| 1st | Pan de Azúcar | 7 | 1.5 | 1.5 | 2.7 |
| 1st | Pan de Azúcar | 7 | 4.3 | 2.5 | 5.5 |
| 1st | Pan de Azúcar | 7 | 0.8 | 1.5 | 1.6 |
| 1st | Pan de Azúcar | 7 | 1.2 | 1 | 2.7 |
| 1st | Pan de Azúcar | 7 | 1 | 1 | 1.2 |
| 1st | Pan de Azúcar | 7 | 1 | 1.1 | 1.3 |
| 1st | Pan de Azúcar | 8 | 0.8 | 0.6 | 1.2 |
| 1st | Pan de Azúcar | 8 | 1.3 | 0.9 | 1.5 |
| 1st | Pan de Azúcar | 8 | 0.9 | 0.7 | 0.8 |
| 1st | Pan de Azúcar | 8 | 1.2 | 1 | 1.3 |
| 1st | Pan de Azúcar | 8 | 1.3 | 1.5 | 2.4 |
| 1st | Pan de Azúcar | 8 | 1 | 2.4 | 1.5 |
| 1st | Pan de Azúcar | 8 | 0.8 | 1 | 1.6 |
| 1st | Pan de Azúcar | 8 | 3 | 3.5 | 2 |
| 1st | Pan de Azúcar | 8 | 3.5 | 2.5 | 3.1 |
| 1st | Pan de Azúcar | 8 | 0.6 | 0.9 | 2.8 |
| 1st | Pan de Azúcar | 8 | 0.6 | 0.9 | 1.6 |
| 1st | Pan de Azúcar | 8 | 2 | 2 | 4 |
| 1st | Pan de Azúcar | 8 | 1 | 1 | 1.9 |
| 1st | Pan de Azúcar | 8 | 2 | 2 | 1.7 |
| 1st | Pan de Azúcar | 8 | 1.5 | 2 | 4 |
| 1st | Pan de Azúcar | 8 | 0.8 | 0.4 | 2.2 |
| 1st | Pan de Azúcar | 8 | 0.4 | 0.6 | 1.5 |
| 1st | Pan de Azúcar | 8 | 0.4 | 0.5 | 0.8 |
| 1st | Pan de Azúcar | 8 | 2 | 2.5 | 3.3 |
| 1st | Pan de Azúcar | 8 | 1.2 | 2 | 1.9 |
| 1st | Pan de Azúcar | 8 | 1.5 | 2 | 3.1 |
| 1st | Pan de Azúcar | 8 | 0.8 | 0.8 | 1.9 |
| 1st | Pan de Azúcar | 8 | 0.7 | 1 | 2 |
| 1st | Pan de Azúcar | 8 | 3.8 | 3.5 | 4.5 |
| 1st | Pan de Azúcar | 8 | 1.8 | 1.8 | 2.6 |
| 1st | Pan de Azúcar | 8 | 2 | 1.7 | 3.7 |
| 1st | Pan de Azúcar | 8 | 2.1 | 1.7 | 2.7 |
| 1st | Pan de Azúcar | 8 | 0.8 | 0.8 | 2.1 |
| 1st | Pan de Azúcar | 8 | 2 | 1.5 | 3.5 |
| 1st | Pan de Azúcar | 9 | 1.8 | 1.8 | 1.9 |
| 1st | Pan de Azúcar | 9 | 5.5 | 4 | 4 |
| 1st | Pan de Azúcar | 9 | 6 | 3 | 4 |
| 1st | Pan de Azúcar | 9 | 3.5 | 3.5 | 5 |
| 1st | Pan de Azúcar | 9 | 2 | 2.5 | 3.5 |
| 1st | Pan de Azúcar | 9 | 2 | 1.5 | 4 |
| 1st | Pan de Azúcar | 9 | 2.5 | 2.5 | 2.5 |
| 1st | Pan de Azúcar | 9 | 0.5 | 0.5 | 2 |
| 1st | Pan de Azúcar | 9 | 0.4 | 1 | 1.8 |
| 1st | Pan de Azúcar | 9 | 1.8 | 2 | 3.4 |
| 1st | Pan de Azúcar | 9 | 1 | 1 | 2.3 |
| 1st | Pan de Azúcar | 9 | 2 | 1 | 2.9 |
| 1st | Pan de Azúcar | 9 | 0.6 | 0.6 | 2 |
| 1st | Pan de Azúcar | 9 | 2 | 1.5 | 4 |
| 1st | Pan de Azúcar | 9 | 2 | 1.5 | 2.5 |
| 1st | Pan de Azúcar | 9 | 1.2 | 1 | 2.1 |
| 1st | Pan de Azúcar | 9 | 2 | 1.5 | 1.9 |
| 1st | Pan de Azúcar | 9 | 2 | 1.5 | 2.3 |
| 1st | Pan de Azúcar | 9 | 4 | 3.5 | 5 |
| 1st | Pan de Azúcar | 9 | 3.5 | 3 | 4 |
| 1st | Pan de Azúcar | 9 | 3.5 | 4.5 | 2.5 |
| 1st | Pan de Azúcar | 9 | 1.8 | 2 | 3.1 |
| 1st | Pan de Azúcar | 9 | 1.5 | 1.5 | 2.6 |
| 1st | Pan de Azúcar | 9 | 2.5 | 2.5 | 3 |
| 1st | Pan de Azúcar | 9 | 1.5 | 1.5 | 1.6 |
| 1st | Pan de Azúcar | 9 | 3.5 | 2.5 | 6.7 |
| 1st | Pan de Azúcar | 9 | 3 | 2.5 | 2.5 |
| 1st | Pan de Azúcar | 9 | 4.5 | 4.5 | 4 |
| 1st | Pan de Azúcar | 9 | 1 | 0.8 | 3.6 |
| 1st | Pan de Azúcar | 9 | 1 | 1.5 | 1.7 |
| 1st | Pan de Azúcar | 9 | 4 | 5.5 | 4 |
| 1st | Pan de Azúcar | 10 | 1 | 1 | 0.5 |
| 1st | Pan de Azúcar | 10 | 2.5 | 3.5 | 3 |
| 1st | Pan de Azúcar | 10 | 1.4 | 1 | 2 |
| 1st | Pan de Azúcar | 10 | 0.8 | 0.7 | 1 |
| 1st | Pan de Azúcar | 10 | 0.8 | 0.9 | 0.9 |
| 1st | Pan de Azúcar | 10 | 0.6 | 0.6 | 1.8 |
| 1st | Pan de Azúcar | 10 | 1 | 0.7 | 0.8 |
| 1st | Pan de Azúcar | 10 | 2 | 2 | 4.2 |
| 1st | Pan de Azúcar | 10 | 1.5 | 1.8 | 2 |
| 1st | Pan de Azúcar | 10 | 1.5 | 1.5 | 2.5 |
| 1st | Pan de Azúcar | 10 | 2 | 3 | 4.9 |
| 1st | Pan de Azúcar | 10 | 1 | 0.5 | 3 |
| 1st | Pan de Azúcar | 10 | 1 | 0.9 | 2.3 |
| 1st | Pan de Azúcar | 10 | 2 | 2.5 | 3.7 |
| 1st | Pan de Azúcar | 11 | 1.2 | 1.5 | 0.3 |
| 1st | Pan de Azúcar | 11 | 0.5 | 0.9 | 0.8 |
| 1st | Pan de Azúcar | 11 | 0.7 | 0.7 | 0.7 |
| 1st | Pan de Azúcar | 11 | 1 | 1.2 | 1.9 |
| 1st | Pan de Azúcar | 11 | 1 | 1.5 | 1.5 |
| 1st | Pan de Azúcar | 11 | 1 | 1.1 | 1 |
| 1st | Pan de Azúcar | 11 | 1.8 | 3 | 2.7 |
| 1st | Pan de Azúcar | 11 | 0.5 | 0.5 | 1.9 |
| 1st | Pan de Azúcar | 11 | 0.5 | 0.6 | 1.1 |
| 1st | Pan de Azúcar | 11 | 0.7 | 1.5 | 1.3 |
| 1st | Pan de Azúcar | 11 | 0.7 | 0.8 | 0.9 |
| 1st | Pan de Azúcar | 11 | 1.2 | 1 | 3.5 |
| 1st | Pan de Azúcar | 11 | 3 | 2 | 3.5 |
| 1st | Pan de Azúcar | 11 | 1 | 0.9 | 0.9 |
| 1st | Pan de Azúcar | 11 | 3.5 | 5.7 | 14 |
| 1st | Pan de Azúcar | 11 | 0.9 | 0.9 | 1.6 |
| 1st | Pan de Azúcar | 11 | 1 | 1 | 4.3 |
| 1st | Pan de Azúcar | 11 | 1 | 0.8 | 3.3 |
| 1st | Pan de Azúcar | 11 | 0.9 | 1.1 | 3.4 |
| 1st | Pan de Azúcar | 11 | 1.5 | 2.5 | 2.5 |
| 1st | Pan de Azúcar | 11 | 0.7 | 0.9 | 2 |
| 1st | Pan de Azúcar | 11 | 0.9 | 0.3 | 1.3 |
| 1st | Pan de Azúcar | 11 | 0.8 | 1.3 | 2.7 |
| 1st | Pan de Azúcar | 11 | 0.7 | 0.7 | 1.3 |
| 1st | Pan de Azúcar | 11 | 0.6 | 0.6 | 0.6 |
| 1st | Pan de Azúcar | 12 | 0.7 | 1 | 1.2 |
| 1st | Pan de Azúcar | 12 | 0.7 | 0.5 | 2 |
| 1st | Pan de Azúcar | 12 | 0.5 | 0.5 | 1.9 |
| 1st | Pan de Azúcar | 12 | 1 | 0.8 | 1.7 |
| 1st | Pan de Azúcar | 12 | 0.9 | 1 | 1.3 |
| 1st | Pan de Azúcar | 12 | 1.2 | 1.1 | 4.5 |
| 1st | Pan de Azúcar | 12 | 1.2 | 1.1 | 1.9 |
| 1st | Pan de Azúcar | 12 | 1 | 1.8 | 3.4 |
| 1st | Pan de Azúcar | 12 | 0.9 | 1.5 | 2.2 |
| 1st | Pan de Azúcar | 12 | 0.5 | 1 | 1.4 |
| 1st | Pan de Azúcar | 12 | 2 | 1.5 | 1.9 |
| 1st | Pan de Azúcar | 12 | 0.5 | 0.6 | 2.5 |
| 1st | Pan de Azúcar | 12 | 0.8 | 1.4 | 1.6 |
| 1st | Pan de Azúcar | 12 | 1 | 1.8 | 1.2 |
| 1st | Pan de Azúcar | 12 | 0.6 | 0.8 | 1 |
| 1st | Pan de Azúcar | 12 | 0.7 | 1 | 0.7 |
| 1st | Pan de Azúcar | 12 | 0.9 | 1 | 1 |
| 1st | Pan de Azúcar | 12 | 2.8 | 3.9 | 10.4 |
| 1st | Santa Gracia | 1 | 10.8 | 4.9 | 5.6 |
| 1st | Santa Gracia | 1 | 10.5 | 3.8 | 7.8 |
| 1st | Santa Gracia | 1 | 6.3 | 3 | 5.4 |
| 1st | Santa Gracia | 1 | 4.5 | 4.2 | 22.9 |
| 1st | Santa Gracia | 1 | 6.3 | 6.3 | 11.9 |
| 1st | Santa Gracia | 1 | 5.8 | 5.3 | 9 |
| 1st | Santa Gracia | 1 | 9.9 | 6.3 | 15.5 |
| 1st | Santa Gracia | 1 | 5 | 5 | 14.2 |
| 1st | Santa Gracia | 1 | 9.7 | 7.7 | 20.3 |
| 1st | Santa Gracia | 1 | 4.6 | 3.9 | 5.7 |
| 1st | Santa Gracia | 1 | 6.9 | 5.2 | 6.1 |
| 1st | Santa Gracia | 1 | 6 | 5.8 | 19.1 |
| 1st | Santa Gracia | 1 | 6.4 | 4.8 | 7.7 |
| 1st | Santa Gracia | 1 | 8.3 | 4.1 | 7.8 |
| 1st | Santa Gracia | 1 | 6 | 4.2 | 5.5 |
| 1st | Santa Gracia | 1 | 8 | 5 | 5.5 |
| 1st | Santa Gracia | 1 | 7.6 | 5.5 | 8 |
| 1st | Santa Gracia | 1 | 10.7 | 5.1 | 6.5 |
| 1st | Santa Gracia | 1 | 10.1 | 8.6 | 8.5 |
| 1st | Santa Gracia | 1 | 14.7 | 9.3 | 17.5 |
| 1st | Santa Gracia | 1 | 8.6 | 9.2 | 11 |
| 1st | Santa Gracia | 1 | 9.5 | 4.6 | 16.5 |
| 1st | Santa Gracia | 1 | 12.1 | 8.4 | 8.5 |
| 1st | Santa Gracia | 1 | 11.2 | 8 | 8.5 |
| 1st | Santa Gracia | 1 | 8.2 | 6.1 | 24.5 |
| 1st | Santa Gracia | 1 | 6 | 6.8 | 6 |
| 1st | Santa Gracia | 1 | 6 | 5.5 | 4 |
| 1st | Santa Gracia | 1 | 8.5 | 4.3 | 8 |
| 1st | Santa Gracia | 1 | 7.2 | 5.1 | 3 |
| 1st | Santa Gracia | 1 | 7.8 | 7.1 | 8 |
| 1st | Santa Gracia | 1 | 14 | 10.5 | 4.5 |
| 1st | Santa Gracia | 1 | 7.3 | 7.3 | 14 |
| 1st | Santa Gracia | 1 | 8 | 11.4 | 10.5 |
| 1st | Santa Gracia | 1 | 3.3 | 3 | 3.5 |
| 1st | Santa Gracia | 1 | 11.6 | 10.8 | 12 |
| 1st | Santa Gracia | 1 | 8.9 | 9.7 | 14.5 |
| 1st | Santa Gracia | 1 | 7.9 | 6.2 | 9.5 |
| 1st | Santa Gracia | 1 | 6.3 | 4.2 | 21 |
| 1st | Santa Gracia | 1 | 7.2 | 6.8 | 18 |
| 1st | Santa Gracia | 1 | 17.2 | 11.6 | 10.5 |
| 1st | Santa Gracia | 1 | 13 | 6.5 | 12.5 |
| 1st | Santa Gracia | 1 | 10 | 6.9 | 10 |
| 1st | Santa Gracia | 1 | 8.2 | 4.7 | 12.5 |
| 1st | Santa Gracia | 1 | 10.8 | 8.3 | 15 |
| 1st | Santa Gracia | 1 | 7.6 | 7.8 | 11 |
| 1st | Santa Gracia | 1 | 6.8 | 5.3 | 6.5 |
| 1st | Santa Gracia | 1 | 5.8 | 7.7 | 18 |
| 1st | Santa Gracia | 1 | 7.1 | 8.2 | 7 |
| 1st | Santa Gracia | 1 | 4.6 | 3.8 | 10 |
| 1st | Santa Gracia | 1 | 4 | 3.1 | 5 |
| 1st | Santa Gracia | 1 | 12 | 9 | 12 |
| 1st | Santa Gracia | 1 | 6.3 | 5.9 | 16 |
| 1st | Santa Gracia | 1 | 9.7 | 7.4 | 9.5 |
| 1st | Santa Gracia | 1 | 6.4 | 9.9 | 15.5 |
| 1st | Santa Gracia | 1 | 8.6 | 9.4 | 18.5 |
| 1st | Santa Gracia | 1 | 7.5 | 5.9 | 7 |
| 1st | Santa Gracia | 1 | 6.5 | 6.8 | 11 |
| 1st | Santa Gracia | 1 | 7.8 | 6.3 | 16 |
| 1st | Santa Gracia | 1 | 6.7 | 7.6 | 12.5 |
| 1st | Santa Gracia | 1 | 1.6 | 0.8 | 2 |
| 1st | Santa Gracia | 1 | 12.4 | 9.2 | 8.5 |
| 1st | Santa Gracia | 1 | 11.2 | 7.4 | 5.5 |
| 1st | Santa Gracia | 1 | 12.3 | 12 | 5 |
| 1st | Santa Gracia | 1 | 8.5 | 7.3 | 9 |
| 1st | Santa Gracia | 1 | 9.4 | 6 | 9.5 |
| 1st | Santa Gracia | 1 | 5.4 | 3.2 | 9 |
| 1st | Santa Gracia | 1 | 5.4 | 5.6 | 10 |
| 1st | Santa Gracia | 1 | 7.7 | 5.9 | 11.5 |
| 1st | Santa Gracia | 1 | 9.4 | 7.2 | 10 |
| 1st | Santa Gracia | 1 | 8.9 | 5.6 | 11.5 |
| 1st | Santa Gracia | 2 | 2 | 3.5 | 5.6 |
| 1st | Santa Gracia | 2 | 3.5 | 3.5 | 7.5 |
| 1st | Santa Gracia | 2 | 3 | 3.5 | 11.5 |
| 1st | Santa Gracia | 2 | 0.5 | 0.9 | 1.1 |
| 1st | Santa Gracia | 2 | 0.4 | 0.5 | 2.5 |
| 1st | Santa Gracia | 2 | 0.6 | 0.7 | 3.2 |
| 1st | Santa Gracia | 2 | 0.7 | 0.8 | 4.1 |
| 1st | Santa Gracia | 2 | 3 | 2 | 3 |
| 1st | Santa Gracia | 2 | 1 | 1 | 6.4 |
| 1st | Santa Gracia | 2 | 0.9 | 1 | 4.6 |
| 1st | Santa Gracia | 2 | 1.5 | 3 | 4 |
| 1st | Santa Gracia | 2 | 0.7 | 0.7 | 1.5 |
| 1st | Santa Gracia | 2 | 1 | 0.8 | 3.2 |
| 1st | Santa Gracia | 2 | 0.3 | 0.5 | 0.7 |
| 1st | Santa Gracia | 2 | 0.6 | 0.4 | 1.6 |
| 1st | Santa Gracia | 2 | 0.7 | 0.7 | 1.1 |
| 1st | Santa Gracia | 2 | 0.5 | 0.5 | 1 |
| 1st | Santa Gracia | 2 | 0.5 | 0.5 | 1.1 |
| 1st | Santa Gracia | 2 | 0.6 | 0.4 | 3.2 |
| 1st | Santa Gracia | 2 | 0.5 | 1.3 | 1.1 |
| 1st | Santa Gracia | 2 | 0.6 | 0.8 | 0.9 |
| 1st | Santa Gracia | 2 | 1.5 | 1.5 | 3 |
| 1st | Santa Gracia | 2 | 2.5 | 3 | 3.5 |
| 1st | Santa Gracia | 2 | 3 | 3 | 6 |
| 1st | Santa Gracia | 2 | 1 | 1 | 3.5 |
| 1st | Santa Gracia | 2 | 4.5 | 7 | 4.5 |
| 1st | Santa Gracia | 2 | 0.5 | 0.5 | 5.5 |
| 1st | Santa Gracia | 2 | 1 | 1.2 | 1.1 |
| 1st | Santa Gracia | 2 | 0.5 | 0.8 | 1.8 |
| 1st | Santa Gracia | 2 | 0.3 | 0.5 | 1.2 |
| 1st | Santa Gracia | 2 | 1.5 | 1.5 | 5.5 |
| 1st | Santa Gracia | 2 | 0.4 | 0.4 | 1.8 |
| 1st | Santa Gracia | 2 | 4 | 2 | 3.5 |
| 1st | Santa Gracia | 2 | 1.5 | 1 | 1.2 |
| 1st | Santa Gracia | 2 | 1.5 | 1 | 1.5 |
| 1st | Santa Gracia | 2 | 1 | 0.9 | 5.3 |
| 1st | Santa Gracia | 2 | 1.5 | 1.3 | 1.8 |
| 1st | Santa Gracia | 2 | 1.5 | 2 | 1 |
| 1st | Santa Gracia | 2 | 1.5 | 1.2 | 1.4 |
| 1st | Santa Gracia | 2 | 3 | 5 | 12 |
| 1st | Santa Gracia | 3 | 1.3 | 1.5 | 1.9 |
| 1st | Santa Gracia | 3 | 1.8 | 2.5 | 5.5 |
| 1st | Santa Gracia | 3 | 1 | 1.2 | 3 |
| 1st | Santa Gracia | 3 | 1.5 | 0.7 | 2.5 |
| 1st | Santa Gracia | 3 | 8.5 | 9 | 6.5 |
| 1st | Santa Gracia | 3 | 3 | 3.5 | 3 |
| 1st | Santa Gracia | 3 | 6.5 | 7 | 6 |
| 1st | Santa Gracia | 3 | 6.5 | 5.5 | 4 |
| 1st | Santa Gracia | 3 | 4.5 | 6.5 | 8 |
| 1st | Santa Gracia | 3 | 0.6 | 0.6 | 3 |
| 1st | Santa Gracia | 4 | 1 | 1 | 2.4 |
| 1st | Santa Gracia | 4 | 2.5 | 3 | 2.3 |
| 1st | Santa Gracia | 4 | 2 | 1.6 | 3.4 |
| 1st | Santa Gracia | 4 | 1.8 | 0.6 | 2.7 |
| 1st | Santa Gracia | 4 | 2 | 3.2 | 1.3 |
| 1st | Santa Gracia | 4 | 1 | 0.9 | 1.1 |
| 1st | Santa Gracia | 4 | 4 | 3 | 4 |
| 1st | Santa Gracia | 4 | 1.3 | 1.7 | 4.9 |
| 1st | Santa Gracia | 4 | 0.8 | 0.7 | 2 |
| 1st | Santa Gracia | 4 | 2 | 2.5 | 2.1 |
| 1st | Santa Gracia | 4 | 1.7 | 1.5 | 4.5 |
| 1st | Santa Gracia | 4 | 2.1 | 2.3 | 5.8 |
| 1st | Santa Gracia | 4 | 1.5 | 1.2 | 3 |
| 1st | Santa Gracia | 4 | 0.9 | 1.2 | 1.6 |
| 1st | Santa Gracia | 4 | 4 | 6 | 4.5 |
| 1st | Santa Gracia | 4 | 0.7 | 0.8 | 1.8 |
| 1st | Santa Gracia | 4 | 1.5 | 2 | 1.7 |
| 1st | Santa Gracia | 4 | 2.5 | 1.5 | 2.3 |
| 1st | Santa Gracia | 4 | 1.8 | 1.5 | 2 |
| 1st | Santa Gracia | 4 | 1.5 | 6 | 2.5 |
| 1st | Santa Gracia | 4 | 2.25 | 1.9 | 1.7 |
| 1st | Santa Gracia | 4 | 1.3 | 1 | 1.9 |
| 1st | Santa Gracia | 4 | 1.3 | 1.3 | 1.7 |
| 1st | Santa Gracia | 4 | 6.8 | 1.5 | 1.5 |
| 1st | Santa Gracia | 4 | 4.5 | 3 | 3 |
| 1st | Santa Gracia | 4 | 1.9 | 2.2 | 1.5 |
| 1st | Santa Gracia | 5 | 1 | 2 | 3 |
| 1st | Santa Gracia | 5 | 2 | 2.5 | 1.3 |
| 1st | Santa Gracia | 5 | 3 | 3 | 1.5 |
| 1st | Santa Gracia | 5 | 4.5 | 9 | 6 |
| 1st | Santa Gracia | 5 | 5 | 6 | 0.8 |
| 1st | Santa Gracia | 5 | 9 | 9.5 | 7 |
| 1st | Santa Gracia | 5 | 4 | 6 | 5 |
| 1st | Santa Gracia | 5 | 3 | 4 | 4 |
| 1st | Santa Gracia | 5 | 1 | 1 | 2.7 |
| 1st | Santa Gracia | 5 | 1.4 | 0.8 | 0.6 |
| 1st | Santa Gracia | 5 | 12.5 | 10.5 | 5.5 |
| 1st | Santa Gracia | 5 | 1 | 1 | 1.7 |
| 1st | Santa Gracia | 5 | 0.6 | 0.9 | 3.2 |
| 1st | Santa Gracia | 5 | 1 | 1.2 | 3.9 |
| 1st | Santa Gracia | 5 | 3 | 3 | 1.2 |
| 1st | Santa Gracia | 5 | 1.6 | 1 | 1.7 |
| 1st | Santa Gracia | 5 | 3.5 | 7 | 3.5 |
| 1st | Santa Gracia | 5 | 0.8 | 2 | 1.2 |
| 1st | Santa Gracia | 5 | 0.5 | 0.4 | 1.8 |
| 1st | Santa Gracia | 5 | 9 | 10 | 11 |
| 1st | Santa Gracia | 5 | 3.5 | 2.5 | 4 |
| 1st | Santa Gracia | 5 | 5 | 5 | 9 |
| 1st | Santa Gracia | 5 | 4 | 7 | 7 |
| 1st | Santa Gracia | 5 | 5.5 | 7.5 | 6.5 |
| 1st | Santa Gracia | 5 | 3 | 7 | 4.5 |
| 1st | Santa Gracia | 5 | 0.5 | 0.5 | 1.5 |
| 1st | Santa Gracia | 5 | 2 | 4.5 | 8 |
| 1st | Santa Gracia | 5 | 3 | 3.5 | 4 |
| 1st | Santa Gracia | 5 | 3.5 | 3 | 5 |
| 1st | Santa Gracia | 5 | 3 | 3 | 1 |
| 1st | Santa Gracia | 5 | 0.9 | 1 | 2 |
| 1st | Santa Gracia | 5 | 1.5 | 2 | 3.5 |
| 1st | Santa Gracia | 5 | 4.5 | 4 | 7 |
| 1st | Santa Gracia | 5 | 2 | 3.5 | 5 |
| 1st | Santa Gracia | 6 | 2.5 | 3 | 4 |
| 1st | Santa Gracia | 6 | 4.5 | 8.5 | 6 |
| 1st | Santa Gracia | 6 | 1 | 2 | 0.5 |
| 1st | Santa Gracia | 6 | 1.5 | 1 | 1.5 |
| 1st | Santa Gracia | 6 | 3 | 6 | 6.5 |
| 1st | Santa Gracia | 6 | 5 | 4 | 10 |
| 1st | Santa Gracia | 6 | 3 | 4 | 5 |
| 1st | Santa Gracia | 6 | 6.5 | 5.5 | 8 |
| 1st | Santa Gracia | 6 | 4.5 | 5 | 3.5 |
| 1st | Santa Gracia | 6 | 9 | 7 | 9.5 |
| 1st | Santa Gracia | 6 | 7 | 7.5 | 9 |
| 1st | Santa Gracia | 6 | 9.5 | 7 | 9 |
| 1st | Santa Gracia | 6 | 5 | 10 | 5 |
| 1st | Santa Gracia | 6 | 4 | 6 | 8 |
| 1st | Santa Gracia | 6 | 1.4 | 1 | 2.4 |
| 1st | Santa Gracia | 6 | 1.5 | 1 | 3.8 |
| 1st | Santa Gracia | 6 | 1 | 1.5 | 2.8 |
| 1st | Santa Gracia | 6 | 2.4 | 1.4 | 1.5 |
| 1st | Santa Gracia | 6 | 1.5 | 1.3 | 1.2 |
| 1st | Santa Gracia | 6 | 1.5 | 1.5 | 1.8 |
| 1st | Santa Gracia | 6 | 5.5 | 4.5 | 7 |
| 1st | Santa Gracia | 6 | 4 | 8 | 4.5 |
| 1st | Santa Gracia | 6 | 3 | 6 | 3 |
| 1st | Santa Gracia | 6 | 9 | 10 | 4 |
| 1st | Santa Gracia | 6 | 1 | 1.2 | 2.2 |
| 1st | Santa Gracia | 6 | 1 | 1 | 2.3 |
| 1st | Santa Gracia | 6 | 1 | 1 | 2.3 |
| 1st | Santa Gracia | 6 | 0.9 | 0.9 | 2.1 |
| 1st | Santa Gracia | 6 | 1.5 | 1.8 | 1.8 |
| 1st | Santa Gracia | 6 | 1.5 | 1.5 | 2.5 |
| 1st | Santa Gracia | 6 | 1.5 | 1.5 | 1.8 |
| 1st | Santa Gracia | 6 | 0.9 | 1 | 2.6 |
| 1st | Santa Gracia | 6 | 1.5 | 1.5 | 2.2 |
| 1st | Santa Gracia | 6 | 1 | 1.5 | 1.5 |
| 1st | Santa Gracia | 6 | 1.1 | 1.4 | 1.5 |
| 1st | Santa Gracia | 6 | 1.4 | 1.4 | 1.2 |
| 1st | Santa Gracia | 6 | 4 | 3.5 | 5 |
| 1st | Santa Gracia | 6 | 0.9 | 1.2 | 1.7 |
| 1st | Santa Gracia | 6 | 1.2 | 1 | 1.5 |
| 1st | Santa Gracia | 6 | 2.6 | 3.2 | 3.5 |
| 1st | Santa Gracia | 7 | 4.5 | 4 | 7 |
| 1st | Santa Gracia | 7 | 1.5 | 1 | 2.8 |
| 1st | Santa Gracia | 7 | 3 | 6 | 5.5 |
| 1st | Santa Gracia | 7 | 2.5 | 3 | 4 |
| 1st | Santa Gracia | 7 | 1.2 | 1.2 | 0.8 |
| 1st | Santa Gracia | 7 | 0.8 | 1.2 | 2.8 |
| 1st | Santa Gracia | 7 | 3 | 4 | 3.5 |
| 1st | Santa Gracia | 7 | 2 | 3 | 2.5 |
| 1st | Santa Gracia | 7 | 1 | 1.2 | 2.6 |
| 1st | Santa Gracia | 7 | 2.2 | 1.3 | 1.6 |
| 1st | Santa Gracia | 7 | 3.5 | 3.5 | 4 |
| 1st | Santa Gracia | 7 | 2 | 2 | 2.3 |
| 1st | Santa Gracia | 7 | 5 | 4.5 | 5 |
| 1st | Santa Gracia | 7 | 0.8 | 1.1 | 2 |
| 1st | Santa Gracia | 7 | 0.4 | 0.6 | 1.2 |
| 1st | Santa Gracia | 7 | 1.2 | 1 | 1.4 |
| 1st | Santa Gracia | 7 | 1 | 1.4 | 1.5 |
| 1st | Santa Gracia | 7 | 1.4 | 1.4 | 1.6 |
| 1st | Santa Gracia | 7 | 1.2 | 1.5 | 2 |
| 1st | Santa Gracia | 7 | 1.2 | 1.1 | 1.8 |
| 1st | Santa Gracia | 7 | 3 | 3.5 | 3 |
| 1st | Santa Gracia | 7 | 0.6 | 0.7 | 1.4 |
| 1st | Santa Gracia | 8 | 1 | 1.1 | 1.4 |
| 1st | Santa Gracia | 8 | 1.5 | 3 | 2 |
| 1st | Santa Gracia | 8 | 4.5 | 5.5 | 4.5 |
| 1st | Santa Gracia | 8 | 1.5 | 1 | 2.7 |
| 1st | Santa Gracia | 8 | 2.5 | 4 | 3 |
| 1st | Santa Gracia | 8 | 3 | 3 | 3.5 |
| 1st | Santa Gracia | 8 | 1.1 | 1.3 | 1.9 |
| 1st | Santa Gracia | 8 | 0.7 | 0.9 | 1.7 |
| 1st | Santa Gracia | 8 | 0.4 | 0.4 | 1.8 |
| 1st | Santa Gracia | 8 | 3.5 | 2 | 2.2 |
| 1st | Santa Gracia | 9 | 1.3 | 1.7 | 1.8 |
| 1st | Santa Gracia | 9 | 1 | 0.8 | 1.4 |
| 1st | Santa Gracia | 9 | 0.9 | 1.2 | 1.5 |
| 1st | Santa Gracia | 9 | 1.5 | 1.5 | 1 |
| 1st | Santa Gracia | 9 | 3.5 | 2.5 | 3 |
| 1st | Santa Gracia | 9 | 5 | 6.5 | 5 |
| 1st | Santa Gracia | 9 | 1.5 | 1.5 | 1 |
| 1st | Santa Gracia | 9 | 1.2 | 1.5 | 0.8 |
| 1st | Santa Gracia | 9 | 2.5 | 5 | 2.5 |
| 1st | Santa Gracia | 9 | 2.5 | 3 | 2 |
| 1st | Santa Gracia | 9 | 2.5 | 3.5 | 2 |
| 1st | Santa Gracia | 9 | 0.9 | 1.2 | 1.5 |
| 1st | Santa Gracia | 9 | 1.5 | 1.8 | 1.6 |
| 1st | Santa Gracia | 9 | 2.5 | 4 | 7 |
| 1st | Santa Gracia | 9 | 5 | 5 | 3.5 |
| 1st | Santa Gracia | 9 | 4 | 8.5 | 2 |
| 1st | Santa Gracia | 9 | 0.9 | 1 | 1.2 |
| 1st | Santa Gracia | 9 | 1 | 2 | 2.1 |
| 1st | Santa Gracia | 9 | 1.5 | 2 | 1.5 |
| 1st | Santa Gracia | 9 | 1.7 | 1.3 | 1.4 |
| 1st | Santa Gracia | 9 | 1.2 | 1.5 | 1.2 |
| 1st | Santa Gracia | 9 | 1.5 | 1.5 | 2.3 |
| 1st | Santa Gracia | 9 | 1 | 1.3 | 2.4 |
| 1st | Santa Gracia | 9 | 1 | 1.2 | 1.7 |
| 1st | Santa Gracia | 9 | 1.2 | 1.3 | 3 |
| 1st | Santa Gracia | 9 | 1.4 | 1.2 | 0.9 |
| 1st | Santa Gracia | 9 | 2.3 | 1.4 | 2.1 |
| 1st | Santa Gracia | 9 | 4 | 5 | 3.7 |
| 1st | Santa Gracia | 9 | 3 | 3.5 | 5 |
| 1st | Santa Gracia | 9 | 2 | 4 | 3.5 |
| 1st | Santa Gracia | 9 | 4 | 6.5 | 5.5 |
| 1st | Santa Gracia | 10 | 1 | 1 | 3.1 |
| 1st | Santa Gracia | 10 | 0.5 | 1 | 0.9 |
| 1st | Santa Gracia | 10 | 1.5 | 1.5 | 1.8 |
| 1st | Santa Gracia | 10 | 1.5 | 1.7 | 1.3 |
| 1st | Santa Gracia | 10 | 0.6 | 0.6 | 1.5 |
| 1st | Santa Gracia | 10 | 0.3 | 0.3 | 1.3 |
| 1st | Santa Gracia | 10 | 9.5 | 7 | 9 |
| 1st | Santa Gracia | 10 | 3.5 | 3 | 2 |
| 1st | Santa Gracia | 10 | 4 | 4.5 | 3.5 |
| 1st | Santa Gracia | 10 | 3.5 | 3 | 3.5 |
| 1st | Santa Gracia | 10 | 7 | 12 | 10 |
| 1st | Santa Gracia | 10 | 0.9 | 1.5 | 0.6 |
| 1st | Santa Gracia | 10 | 7 | 6.5 | 6.5 |
| 1st | Santa Gracia | 10 | 1.2 | 1.9 | 1.7 |
| 1st | Santa Gracia | 11 | 4 | 2.5 | 3 |
| 1st | Santa Gracia | 11 | 1.1 | 1.5 | 3.1 |
| 1st | Santa Gracia | 11 | 1.5 | 1.7 | 4.8 |
| 1st | Santa Gracia | 11 | 3 | 2.5 | 2.5 |
| 1st | Santa Gracia | 11 | 1.1 | 1.3 | 1.2 |
| 1st | Santa Gracia | 11 | 2 | 2.2 | 2.8 |
| 1st | Santa Gracia | 11 | 0.7 | 0.7 | 2.1 |
| 1st | Santa Gracia | 11 | 1 | 1.5 | 2.2 |
| 1st | Santa Gracia | 11 | 1.6 | 1.6 | 1.1 |
| 1st | Santa Gracia | 11 | 1.5 | 1.7 | 1.6 |
| 1st | Santa Gracia | 11 | 1.2 | 1.3 | 0.7 |
| 1st | Santa Gracia | 11 | 1.5 | 1.9 | 1.3 |
| 1st | Santa Gracia | 11 | 1.8 | 1.9 | 1.8 |
| 1st | Santa Gracia | 11 | 0.6 | 0.7 | 1.3 |
| 1st | Santa Gracia | 12 | 1.2 | 1.3 | 7.7 |
| 1st | Santa Gracia | 12 | 1.4 | 1.2 | 2.9 |
| 1st | Santa Gracia | 12 | 1.6 | 1.5 | 3.7 |
| 1st | Santa Gracia | 12 | 0.4 | 1 | 4 |
| 1st | Santa Gracia | 12 | 2 | 1.8 | 4.3 |
| 1st | Santa Gracia | 12 | 1.6 | 1.8 | 2.9 |
| 1st | Santa Gracia | 12 | 1.1 | 1.2 | 1.7 |
| 1st | Santa Gracia | 12 | 0.8 | 0.9 | 2.6 |
| 1st | Santa Gracia | 12 | 0.6 | 0.5 | 1.9 |
| 1st | Santa Gracia | 12 | 1 | 1.3 | 2 |
| 1st | Santa Gracia | 12 | 3 | 4.5 | 6.5 |
| 1st | Santa Gracia | 12 | 3 | 3.2 | 1.5 |
| 1st | La CampaNahuelbuta | 1 | 3.7 | 3.3 | 1.7 |
| 1st | La CampaNahuelbuta | 1 | 6.9 | 5.5 | 6.5 |
| 1st | La CampaNahuelbuta | 1 | 6.5 | 4.5 | 3 |
| 1st | La CampaNahuelbuta | 2 | 2 | 1.8 | 1 |
| 1st | La CampaNahuelbuta | 2 | 6.5 | 8 | 3 |
| 1st | La CampaNahuelbuta | 2 | 5.5 | 8 | 2 |
| 1st | La CampaNahuelbuta | 2 | 3.5 | 2.5 | 2.3 |
| 1st | La CampaNahuelbuta | 2 | 2.5 | 2 | 6 |
| 1st | La CampaNahuelbuta | 2 | 2.7 | 4.5 | 4 |
| 1st | La CampaNahuelbuta | 2 | 1.3 | 3 | 1.5 |
| 1st | La CampaNahuelbuta | 2 | 4.5 | 6 | 1.7 |
| 1st | La CampaNahuelbuta | 2 | 5 | 7 | 3.5 |
| 1st | La CampaNahuelbuta | 2 | 5 | 6.5 | 7 |
| 1st | La CampaNahuelbuta | 2 | 5.5 | 6.5 | 4.5 |
| 1st | La CampaNahuelbuta | 3 | 0.5 | 0.7 | 1.2 |
| 1st | La CampaNahuelbuta | 3 | 0.8 | 1.4 | 0.7 |
| 1st | La CampaNahuelbuta | 3 | 12 | 6 | 5 |
| 1st | La CampaNahuelbuta | 3 | 10 | 11 | 11 |
| 1st | La CampaNahuelbuta | 3 | 10 | 8 | 1.5 |
| 1st | La CampaNahuelbuta | 3 | 0.8 | 0.8 | 1.2 |
| 1st | La CampaNahuelbuta | 3 | 4.5 | 2.5 | 1 |
| 1st | La CampaNahuelbuta | 3 | 5 | 3.5 | 4 |
| 1st | La CampaNahuelbuta | 4 | 1.2 | 1.5 | 0.6 |
| 1st | La CampaNahuelbuta | 4 | 0.9 | 1 | 0.9 |
| 1st | La CampaNahuelbuta | 4 | 0.5 | 0.6 | 3.2 |
| 1st | La CampaNahuelbuta | 4 | 0.7 | 0.7 | 0.9 |
| 1st | La CampaNahuelbuta | 5 | 3 | 9 | 2 |
| 1st | La CampaNahuelbuta | 5 | 9 | 6.5 | 8.5 |
| 1st | La CampaNahuelbuta | 5 | 0.7 | 0.7 | 1 |
| 1st | La CampaNahuelbuta | 5 | 0.6 | 0.6 | 1.6 |
| 1st | La CampaNahuelbuta | 5 | 0.8 | 0.7 | 4.9 |
| 1st | La CampaNahuelbuta | 5 | 0.6 | 0.4 | 1.4 |
| 1st | La CampaNahuelbuta | 6 | 0.3 | 0.3 | 0.5 |
| 1st | La CampaNahuelbuta | 6 | 0.8 | 0.2 | 0.9 |
| 1st | La CampaNahuelbuta | 6 | 0.6 | 0.6 | 1.2 |
| 1st | La CampaNahuelbuta | 7 | 12.5 | 10 | 12 |
| 1st | La CampaNahuelbuta | 7 | 7.5 | 6 | 3 |
| 1st | La CampaNahuelbuta | 7 | 3 | 4 | 1.5 |
| 1st | La CampaNahuelbuta | 7 | 5.5 | 6.5 | 3 |
| 1st | La CampaNahuelbuta | 7 | 7 | 3.5 | 5 |
| 1st | La CampaNahuelbuta | 7 | 3.5 | 2 | 2 |
| 1st | La CampaNahuelbuta | 7 | 2.5 | 4 | 3 |
| 1st | La CampaNahuelbuta | 7 | 0.8 | 0.6 | 0.7 |
| 1st | La CampaNahuelbuta | 7 | 3 | 2 | 2 |
| 1st | La CampaNahuelbuta | 7 | 1.5 | 1.6 | 1.7 |
| 1st | La CampaNahuelbuta | 7 | 2.5 | 1.5 | 1.5 |
| 1st | La CampaNahuelbuta | 8 | 14.5 | 12 | 5 |
| 1st | La CampaNahuelbuta | 9 | 0.6 | 0.6 | 1.8 |
| 1st | La CampaNahuelbuta | 9 | 0.8 | 0.7 | 1.3 |
| 1st | La CampaNahuelbuta | 9 | 2 | 2.2 | 9 |
| 1st | La CampaNahuelbuta | 9 | 3 | 5 | 2 |
| 1st | La CampaNahuelbuta | 9 | 3 | 2.5 | 1.5 |
| 1st | La CampaNahuelbuta | 9 | 3 | 4.5 | 1.5 |
| 1st | La CampaNahuelbuta | 9 | 1 | 1.3 | 1 |
| 1st | La CampaNahuelbuta | 10 | 2.5 | 3 | 1.7 |
| 1st | La CampaNahuelbuta | 10 | 6 | 6.5 | 3 |
| 1st | La CampaNahuelbuta | 10 | 3.5 | 6 | 1.5 |
| 1st | La CampaNahuelbuta | 10 | 6 | 4 | 5 |
| 1st | La CampaNahuelbuta | 10 | 8 | 14 | 5 |
| 1st | La CampaNahuelbuta | 10 | 6 | 9 | 6 |
| 1st | La CampaNahuelbuta | 10 | 5 | 4 | 2 |
| 1st | La CampaNahuelbuta | 10 | 5 | 6 | 3 |
| 1st | La CampaNahuelbuta | 10 | 8 | 12 | 3 |
| 1st | La CampaNahuelbuta | 10 | 5 | 4.5 | 3.5 |
| 1st | La CampaNahuelbuta | 10 | 7 | 4 | 5 |
| 1st | La CampaNahuelbuta | 10 | 5.5 | 4 | 5 |
| 1st | La CampaNahuelbuta | 10 | 8 | 8 | 2 |
| 1st | La CampaNahuelbuta | 10 | 9 | 9 | 5 |
| 1st | La CampaNahuelbuta | 10 | 5 | 4.5 | 5 |
| 1st | La CampaNahuelbuta | 10 | 3.5 | 3 | 6.5 |
| 1st | La CampaNahuelbuta | 10 | 8 | 4 | 6 |
| 1st | La CampaNahuelbuta | 10 | 6.5 | 6 | 2 |
| 1st | La CampaNahuelbuta | 10 | 4.5 | 5.5 | 8 |
| 1st | La CampaNahuelbuta | 10 | 1.2 | 1 | 0.4 |
| 1st | La CampaNahuelbuta | 10 | 5.5 | 3 | 1 |
| 1st | La CampaNahuelbuta | 10 | 5 | 4 | 4.5 |
| 1st | La CampaNahuelbuta | 10 | 3 | 3 | 2.3 |
| 1st | La CampaNahuelbuta | 10 | 11 | 7 | 6 |
| 1st | La CampaNahuelbuta | 10 | 5.5 | 3.5 | 3 |
| 1st | La CampaNahuelbuta | 10 | 5 | 4.5 | 13 |
| 1st | La CampaNahuelbuta | 10 | 0.8 | 2.4 | 0.8 |
| 1st | La CampaNahuelbuta | 10 | 5 | 5 | 6 |
| 1st | La CampaNahuelbuta | 10 | 6.5 | 10 | 9 |
| 1st | La CampaNahuelbuta | 10 | 6 | 5 | 8 |
| 1st | La CampaNahuelbuta | 10 | 5 | 5 | 7.5 |
| 1st | La CampaNahuelbuta | 10 | 5.5 | 9 | 4.5 |
| 1st | La CampaNahuelbuta | 10 | 4.5 | 5 | 0.8 |
| 1st | La CampaNahuelbuta | 10 | 4 | 3 | 2.1 |
| 1st | La CampaNahuelbuta | 10 | 3 | 5.5 | 6 |
| 1st | La CampaNahuelbuta | 10 | 1.5 | 2 | 1.6 |
| 1st | La CampaNahuelbuta | 10 | 7 | 9 | 8 |
| 1st | La CampaNahuelbuta | 10 | 3 | 3 | 1.3 |
| 1st | La CampaNahuelbuta | 10 | 1.5 | 3 | 1.6 |
| 1st | La CampaNahuelbuta | 10 | 10.5 | 15 | 7 |
| 1st | La CampaNahuelbuta | 11 | 22.5 | 13 | 15 |
| 1st | La CampaNahuelbuta | 11 | 12.5 | 11.5 | 13.5 |
| 1st | La CampaNahuelbuta | 11 | 8 | 8.5 | 4 |
| 1st | La CampaNahuelbuta | 11 | 1.5 | 4 | 1.5 |
| 1st | La CampaNahuelbuta | 11 | 4.5 | 5 | 3 |
| 1st | La CampaNahuelbuta | 11 | 1.5 | 2 | 0.5 |
| 1st | La CampaNahuelbuta | 11 | 1.5 | 2 | 0.6 |
| 1st | La CampaNahuelbuta | 11 | 4.5 | 5 | 4.5 |
| 1st | La CampaNahuelbuta | 11 | 3 | 3.5 | 2 |
| 1st | La CampaNahuelbuta | 11 | 1.5 | 2 | 1.2 |
| 1st | La CampaNahuelbuta | 11 | 1.5 | 1.5 | 1.7 |
| 1st | La CampaNahuelbuta | 11 | 2 | 2 | 1.4 |
| 1st | La CampaNahuelbuta | 11 | 43 | 26.5 | 39 |
| 1st | La CampaNahuelbuta | 11 | 5.5 | 8 | 2.5 |
| 1st | La CampaNahuelbuta | 11 | 9 | 7 | 8 |
| 1st | La CampaNahuelbuta | 11 | 3 | 4.5 | 7 |
| 1st | La CampaNahuelbuta | 11 | 6 | 6 | 2.5 |
| 1st | La CampaNahuelbuta | 11 | 5 | 6 | 9 |
| 1st | La CampaNahuelbuta | 11 | 2 | 3 | 4.5 |
| 1st | La CampaNahuelbuta | 11 | 12.5 | 17.5 | 10 |
| 1st | La CampaNahuelbuta | 12 | 0.6 | 0.6 | 1.9 |
| 1st | La CampaNahuelbuta | 12 | 0.6 | 0.4 | 0.6 |
| 1st | La CampaNahuelbuta | 12 | 0.3 | 1 | 1.2 |
| 1st | La CampaNahuelbuta | 12 | 1 | 0.9 | 2.6 |
| 1st | La CampaNahuelbuta | 12 | 0.3 | 0.5 | 0.9 |
| 1st | La CampaNahuelbuta | 12 | 0.5 | 0.5 | 1.1 |
| 1st | La CampaNahuelbuta | 12 | 0.7 | 0.8 | 1.2 |
| 1st | La CampaNahuelbuta | 12 | 0.5 | 0.6 | 2.4 |
| 1st | La CampaNahuelbuta | 12 | 0.9 | 0.7 | 2.8 |
| 1st | La CampaNahuelbuta | 12 | 1 | 1 | 0.9 |
| 1st | La CampaNahuelbuta | 12 | 0.8 | 0.8 | 1.5 |
| 1st | Nahuelbuta | 1 | 7 | 8 | 5 |
| 1st | Nahuelbuta | 1 | 2.5 | 2 | 2.6 |
| 1st | Nahuelbuta | 1 | 2.6 | 2.7 | 1.1 |
| 1st | Nahuelbuta | 1 | 6 | 5.5 | 6.5 |
| 1st | Nahuelbuta | 1 | 14 | 12 | 9 |
| 1st | Nahuelbuta | 1 | 9 | 10 | 9.5 |
| 1st | Nahuelbuta | 1 | 9.5 | 8 | 7.5 |
| 1st | Nahuelbuta | 2 | 0.4 | 0.4 | 1 |
| 1st | Nahuelbuta | 2 | 0.9 | 0.9 | 2.5 |
| 1st | Nahuelbuta | 2 | 1.1 | 1.3 | 5.3 |
| 1st | Nahuelbuta | 2 | 0.9 | 0.8 | 3.4 |
| 1st | Nahuelbuta | 2 | 0.4 | 0.4 | 1.7 |
| 1st | Nahuelbuta | 2 | 0.3 | 0.4 | 2.2 |
| 1st | Nahuelbuta | 2 | 0.5 | 0.5 | 0.9 |
| 1st | Nahuelbuta | 2 | 0.2 | 0.2 | 0.9 |
| 1st | Nahuelbuta | 2 | 0.2 | 0.5 | 1.1 |
| 1st | Nahuelbuta | 2 | 0.4 | 0.6 | 1.6 |
| 1st | Nahuelbuta | 2 | 0.5 | 0.5 | 0.6 |
| 1st | Nahuelbuta | 2 | 0.8 | 0.6 | 0.8 |
| 1st | Nahuelbuta | 2 | 0.3 | 0.3 | 1.5 |
| 1st | Nahuelbuta | 2 | 1.1 | 1 | 1.7 |
| 1st | Nahuelbuta | 2 | 1.5 | 1.5 | 2.3 |
| 1st | Nahuelbuta | 3 | 10 | 9 | 5 |
| 1st | Nahuelbuta | 3 | 9 | 7 | 11 |
| 1st | Nahuelbuta | 3 | 2 | 3 | 5 |
| 1st | Nahuelbuta | 3 | 8 | 12 | 8 |
| 1st | Nahuelbuta | 3 | 8 | 8 | 6.5 |
| 1st | Nahuelbuta | 3 | 13 | 9 | 6 |
| 1st | Nahuelbuta | 3 | 14.5 | 11 | 8.5 |
| 1st | Nahuelbuta | 3 | 7.5 | 12 | 11 |
| 1st | Nahuelbuta | 3 | 9 | 11 | 10 |
| 1st | Nahuelbuta | 3 | 10 | 9.5 | 9 |
| 1st | Nahuelbuta | 3 | 7 | 6.5 | 10 |
| 1st | Nahuelbuta | 3 | 7 | 9 | 12 |
| 1st | Nahuelbuta | 3 | 7 | 6.5 | 8 |
| 1st | Nahuelbuta | 3 | 3 | 2.5 | 2 |
| 1st | Nahuelbuta | 3 | 9 | 7 | 10 |
| 1st | Nahuelbuta | 3 | 10 | 13 | 20 |
| 1st | Nahuelbuta | 4 | 0.8 | 1 | 2.2 |
| 1st | Nahuelbuta | 4 | 1.5 | 2 | 2.9 |
| 1st | Nahuelbuta | 4 | 0.8 | 0.7 | 2.3 |
| 1st | Nahuelbuta | 4 | 0.6 | 0.6 | 2.7 |
| 1st | Nahuelbuta | 4 | 0.5 | 0.4 | 3.8 |
| 1st | Nahuelbuta | 4 | 0.6 | 0.7 | 2.9 |
| 1st | Nahuelbuta | 4 | 0.3 | 0.3 | 1.3 |
| 1st | Nahuelbuta | 4 | 2 | 1.8 | 3.6 |
| 1st | Nahuelbuta | 4 | 0.5 | 0.5 | 3.7 |
| 1st | Nahuelbuta | 4 | 2.5 | 1.5 | 0.9 |
| 1st | Nahuelbuta | 4 | 0.6 | 0.6 | 1.8 |
| 1st | Nahuelbuta | 5 | 0.2 | 0.3 | 1.8 |
| 1st | Nahuelbuta | 5 | 0.9 | 1 | 1.6 |
| 1st | Nahuelbuta | 5 | 0.7 | 0.9 | 3.7 |
| 1st | Nahuelbuta | 5 | 1.5 | 1.5 | 2.6 |
| 1st | Nahuelbuta | 5 | 0.4 | 0.5 | 1.7 |
| 1st | Nahuelbuta | 5 | 0.5 | 0.5 | 1.1 |
| 1st | Nahuelbuta | 5 | 6.5 | 8 | 8 |
| 1st | Nahuelbuta | 5 | 0.8 | 0.9 | 2.4 |
| 1st | Nahuelbuta | 5 | 1 | 1.5 | 2.8 |
| 1st | Nahuelbuta | 5 | 1 | 1 | 1.4 |
| 1st | Nahuelbuta | 5 | 0.6 | 0.6 | 6.1 |
| 1st | Nahuelbuta | 5 | 2 | 3 | 10 |
| 1st | Nahuelbuta | 5 | 0.7 | 0.8 | 2.4 |
| 1st | Nahuelbuta | 5 | 2 | 3.5 | 1.5 |
| 1st | Nahuelbuta | 6 | 0.6 | 0.8 | 3.2 |
| 1st | Nahuelbuta | 6 | 0.9 | 0.6 | 1.9 |
| 1st | Nahuelbuta | 6 | 1 | 1 | 3 |
| 1st | Nahuelbuta | 6 | 0.4 | 0.6 | 2 |
| 1st | Nahuelbuta | 6 | 0.2 | 0.2 | 0.9 |
| 1st | Nahuelbuta | 6 | 0.5 | 0.3 | 1.3 |
| 1st | Nahuelbuta | 6 | 1.5 | 3 | 1.7 |
| 1st | Nahuelbuta | 6 | 2 | 3 | 10.4 |
| 1st | Nahuelbuta | 7 | NA | NA | NA |
| 1st | Nahuelbuta | 8 | 3 | 3.5 | 2 |
| 1st | Nahuelbuta | 8 | 2.8 | 6 | 6.5 |
| 1st | Nahuelbuta | 8 | 3 | 4 | 5 |
| 1st | Nahuelbuta | 8 | 9 | 7 | 3.5 |
| 1st | Nahuelbuta | 8 | 5 | 4.5 | 2.5 |
| 1st | Nahuelbuta | 8 | 4 | 5 | 3.5 |
| 1st | Nahuelbuta | 9 | 4 | 7.5 | 5 |
| 1st | Nahuelbuta | 9 | 2 | 3 | 3.5 |
| 1st | Nahuelbuta | 9 | 5 | 4 | 12.5 |
| 1st | Nahuelbuta | 9 | 8 | 5 | 4.5 |
| 1st | Nahuelbuta | 9 | 2 | 2.5 | 2 |
| 1st | Nahuelbuta | 9 | 3 | 3.5 | 1.7 |
| 1st | Nahuelbuta | 9 | 4.5 | 3 | 3 |
| 1st | Nahuelbuta | 10 | NA | NA | NA |
| 1st | Nahuelbuta | 11 | NA | NA | NA |
| 1st | Nahuelbuta | 12 | NA | NA | NA |
| 2nd | Pan de Azúcar | 1 | 1 | 2.5 | 0.7 |
| 2nd | Pan de Azúcar | 1 | 0.8 | 1 | 0.5 |
| 2nd | Pan de Azúcar | 1 | 0.8 | 0.8 | 0.7 |
| 2nd | Pan de Azúcar | 1 | 1.2 | 1.1 | 1 |
| 2nd | Pan de Azúcar | 1 | 1 | 0.8 | 0.3 |
| 2nd | Pan de Azúcar | 1 | 0.5 | 0.6 | 0.2 |
| 2nd | Pan de Azúcar | 1 | 0.7 | 0.4 | 0.3 |
| 2nd | Pan de Azúcar | 1 | 3.2 | 5 | 4.2 |
| 2nd | Pan de Azúcar | 2 | 0.8 | 1 | 0.4 |
| 2nd | Pan de Azúcar | 2 | 0.7 | 1 | 0.3 |
| 2nd | Pan de Azúcar | 2 | 0.5 | 0.5 | 0.2 |
| 2nd | Pan de Azúcar | 2 | 0.6 | 0.5 | 0.2 |
| 2nd | Pan de Azúcar | 2 | 0.8 | 0.7 | 0.2 |
| 2nd | Pan de Azúcar | 2 | 2 | 3.1 | 1.5 |
| 2nd | Pan de Azúcar | 2 | 2.5 | 6 | 4.5 |
| 2nd | Pan de Azúcar | 2 | 1.5 | 1.2 | 1.4 |
| 2nd | Pan de Azúcar | 2 | 0.7 | 0.6 | 0.6 |
| 2nd | Pan de Azúcar | 2 | 0.7 | 0.5 | 0.2 |
| 2nd | Pan de Azúcar | 2 | 0.6 | 0.6 | 0.3 |
| 2nd | Pan de Azúcar | 2 | 0.3 | 0.3 | 0.1 |
| 2nd | Pan de Azúcar | 2 | 0.6 | 0.5 | 0.1 |
| 2nd | Pan de Azúcar | 2 | 0.6 | 1.1 | 0.8 |
| 2nd | Pan de Azúcar | 2 | 0.4 | 0.5 | 0.2 |
| 2nd | Pan de Azúcar | 2 | 1.2 | 1.5 | 1 |
| 2nd | Pan de Azúcar | 2 | 1.2 | 1 | 0.6 |
| 2nd | Pan de Azúcar | 2 | 0.9 | 2 | 0.4 |
| 2nd | Pan de Azúcar | 2 | 0.4 | 0.7 | 0.3 |
| 2nd | Pan de Azúcar | 2 | 0.9 | 0.9 | 0.4 |
| 2nd | Pan de Azúcar | 2 | 0.6 | 0.5 | 0.1 |
| 2nd | Pan de Azúcar | 2 | 0.6 | 0.4 | 0.1 |
| 2nd | Pan de Azúcar | 2 | 0.4 | 0.6 | 0.2 |
| 2nd | Pan de Azúcar | 2 | 1.5 | 1.5 | 1 |
| 2nd | Pan de Azúcar | 2 | 0.4 | 0.5 | 0.3 |
| 2nd | Pan de Azúcar | 2 | 0.4 | 0.3 | 0.1 |
| 2nd | Pan de Azúcar | 2 | 0.6 | 0.6 | 0.6 |
| 2nd | Pan de Azúcar | 3 | 1.5 | 4 | 3.5 |
| 2nd | Pan de Azúcar | 3 | 1.5 | 2 | 2.5 |
| 2nd | Pan de Azúcar | 3 | 1.5 | 2.5 | 1.5 |
| 2nd | Pan de Azúcar | 3 | 2.7 | 2.9 | 1.7 |
| 2nd | Pan de Azúcar | 3 | 2 | 3 | 2.5 |
| 2nd | Pan de Azúcar | 3 | 3 | 2 | 2.5 |
| 2nd | Pan de Azúcar | 3 | 1 | 0.7 | 0.4 |
| 2nd | Pan de Azúcar | 3 | 2 | 3 | 4 |
| 2nd | Pan de Azúcar | 3 | 1 | 3 | 4.5 |
| 2nd | Pan de Azúcar | 3 | 2 | 3 | 2.2 |
| 2nd | Pan de Azúcar | 3 | 6 | 19 | 20 |
| 2nd | Pan de Azúcar | 3 | 0.5 | 0.9 | 1.4 |
| 2nd | Pan de Azúcar | 3 | 3.5 | 7 | 4.7 |
| 2nd | Pan de Azúcar | 4 | 0.8 | 1 | 0.3 |
| 2nd | Pan de Azúcar | 4 | 1 | 0.7 | 0.2 |
| 2nd | Pan de Azúcar | 4 | 0.7 | 1.3 | 0.4 |
| 2nd | Pan de Azúcar | 4 | 0.9 | 1.3 | 0.9 |
| 2nd | Pan de Azúcar | 4 | 0.9 | 1 | 0.6 |
| 2nd | Pan de Azúcar | 4 | 2 | 1.7 | 1.4 |
| 2nd | Pan de Azúcar | 4 | 13 | 13.5 | 7 |
| 2nd | Pan de Azúcar | 4 | 1.5 | 2.5 | 0.4 |
| 2nd | Pan de Azúcar | 4 | 1.3 | 0.8 | 0.4 |
| 2nd | Pan de Azúcar | 4 | 0.7 | 0.7 | 0.5 |
| 2nd | Pan de Azúcar | 4 | 0.6 | 0.9 | 0.7 |
| 2nd | Pan de Azúcar | 4 | 1 | 1 | 0.5 |
| 2nd | Pan de Azúcar | 4 | 0.9 | 1 | 0.5 |
| 2nd | Pan de Azúcar | 4 | 0.8 | 1 | 0.6 |
| 2nd | Pan de Azúcar | 4 | 0.9 | 0.9 | 0.8 |
| 2nd | Pan de Azúcar | 5 | 2 | 0.7 | 0.4 |
| 2nd | Pan de Azúcar | 5 | 1.5 | 2 | 1.3 |
| 2nd | Pan de Azúcar | 5 | 0.8 | 1 | 0.5 |
| 2nd | Pan de Azúcar | 5 | 1 | 1 | 1.4 |
| 2nd | Pan de Azúcar | 5 | 1 | 0.8 | 0.3 |
| 2nd | Pan de Azúcar | 5 | 1 | 0.7 | 0.6 |
| 2nd | Pan de Azúcar | 5 | 1.5 | 1.5 | 0.9 |
| 2nd | Pan de Azúcar | 6 | 1 | 1 | 0.3 |
| 2nd | Pan de Azúcar | 6 | 1.1 | 0.8 | 0.5 |
| 2nd | Pan de Azúcar | 6 | 0.6 | 0.6 | 0.5 |
| 2nd | Pan de Azúcar | 6 | 1 | 0.8 | 0.4 |
| 2nd | Pan de Azúcar | 6 | 2.5 | 3.5 | 1.5 |
| 2nd | Pan de Azúcar | 6 | 1.2 | 1 | 0.9 |
| 2nd | Pan de Azúcar | 7 | 1 | 1 | 0.8 |
| 2nd | Pan de Azúcar | 7 | 1.4 | 2.4 | 0.8 |
| 2nd | Pan de Azúcar | 7 | 1 | 4 | 0.9 |
| 2nd | Pan de Azúcar | 7 | 1 | 2 | 0.2 |
| 2nd | Pan de Azúcar | 7 | 0.4 | 0.5 | 0.3 |
| 2nd | Pan de Azúcar | 7 | 0.4 | 0.5 | 0.3 |
| 2nd | Pan de Azúcar | 7 | 0.6 | 0.6 | 0.3 |
| 2nd | Pan de Azúcar | 7 | 1.2 | 1.5 | 1 |
| 2nd | Pan de Azúcar | 7 | 1 | 1.5 | 0.4 |
| 2nd | Pan de Azúcar | 7 | 0.7 | 1 | 0.2 |
| 2nd | Pan de Azúcar | 7 | 0.9 | 1 | 0.5 |
| 2nd | Pan de Azúcar | 7 | 1.5 | 2 | 0.8 |
| 2nd | Pan de Azúcar | 7 | 0.8 | 1.3 | 0.9 |
| 2nd | Pan de Azúcar | 7 | 2 | 2.5 | 1.3 |
| 2nd | Pan de Azúcar | 7 | 0.9 | 1 | 0.6 |
| 2nd | Pan de Azúcar | 8 | 0.6 | 0.5 | 0.1 |
| 2nd | Pan de Azúcar | 8 | 1 | 1.1 | 1.6 |
| 2nd | Pan de Azúcar | 8 | 2 | 2.3 | 1.9 |
| 2nd | Pan de Azúcar | 8 | 0.9 | 3.3 | 1.5 |
| 2nd | Pan de Azúcar | 8 | 0.8 | 1.1 | 0.1 |
| 2nd | Pan de Azúcar | 8 | 1 | 1.8 | 1.2 |
| 2nd | Pan de Azúcar | 8 | 0.8 | 0.9 | 0.4 |
| 2nd | Pan de Azúcar | 8 | 1.8 | 1.4 | 0.8 |
| 2nd | Pan de Azúcar | 8 | 0.8 | 1 | 0.4 |
| 2nd | Pan de Azúcar | 8 | 0.3 | 0.9 | 0.3 |
| 2nd | Pan de Azúcar | 8 | 0.9 | 1.5 | 0.2 |
| 2nd | Pan de Azúcar | 8 | 0.8 | 0.9 | 0.6 |
| 2nd | Pan de Azúcar | 8 | 0.4 | 0.8 | 0.4 |
| 2nd | Pan de Azúcar | 8 | 2 | 2.2 | 1.2 |
| 2nd | Pan de Azúcar | 8 | 2 | 2.5 | 0.2 |
| 2nd | Pan de Azúcar | 8 | 1 | 1.4 | 0.1 |
| 2nd | Pan de Azúcar | 8 | 0.9 | 0.4 | 0.2 |
| 2nd | Pan de Azúcar | 8 | 0.3 | 0.8 | 0.2 |
| 2nd | Pan de Azúcar | 8 | 0.9 | 0.4 | 0.2 |
| 2nd | Pan de Azúcar | 8 | 1 | 1.9 | 1 |
| 2nd | Pan de Azúcar | 8 | 0.9 | 1 | 0.3 |
| 2nd | Pan de Azúcar | 8 | 0.5 | 1 | 0.7 |
| 2nd | Pan de Azúcar | 8 | 1.5 | 2 | 0.9 |
| 2nd | Pan de Azúcar | 8 | 1.8 | 1.9 | 0.5 |
| 2nd | Pan de Azúcar | 8 | 0.8 | 0.8 | 0.3 |
| 2nd | Pan de Azúcar | 8 | 0.4 | 0.6 | 0.2 |
| 2nd | Pan de Azúcar | 8 | 0.4 | 0.4 | 0.1 |
| 2nd | Pan de Azúcar | 8 | 1.5 | 1 | 0.5 |
| 2nd | Pan de Azúcar | 8 | 1 | 0.6 | 0.3 |
| 2nd | Pan de Azúcar | 9 | 2 | 2.5 | 1 |
| 2nd | Pan de Azúcar | 9 | 0.4 | 0.5 | 0.2 |
| 2nd | Pan de Azúcar | 9 | 1 | 1 | 0.2 |
| 2nd | Pan de Azúcar | 9 | 1 | 1.2 | 0.6 |
| 2nd | Pan de Azúcar | 9 | 1.5 | 1.2 | 0.6 |
| 2nd | Pan de Azúcar | 9 | 1 | 1 | 0.2 |
| 2nd | Pan de Azúcar | 9 | 1 | 1.5 | 0.7 |
| 2nd | Pan de Azúcar | 9 | 0.3 | 1.2 | 0.2 |
| 2nd | Pan de Azúcar | 9 | 2.3 | 2.2 | 1.9 |
| 2nd | Pan de Azúcar | 9 | 2 | 3 | 0.2 |
| 2nd | Pan de Azúcar | 9 | 1.2 | 2 | 0.4 |
| 2nd | Pan de Azúcar | 9 | 0.9 | 0.5 | 0.3 |
| 2nd | Pan de Azúcar | 9 | 1.9 | 1.2 | 0.2 |
| 2nd | Pan de Azúcar | 9 | 2 | 1.4 | 0.4 |
| 2nd | Pan de Azúcar | 9 | 1 | 1.7 | 0.4 |
| 2nd | Pan de Azúcar | 9 | 1.6 | 2.2 | 1.2 |
| 2nd | Pan de Azúcar | 9 | 0.9 | 1 | 0.6 |
| 2nd | Pan de Azúcar | 9 | 1 | 1.8 | 0.6 |
| 2nd | Pan de Azúcar | 9 | 1.6 | 1.9 | 0.2 |
| 2nd | Pan de Azúcar | 9 | 1.2 | 1.3 | 1 |
| 2nd | Pan de Azúcar | 9 | 1 | 1.2 | 0.2 |
| 2nd | Pan de Azúcar | 9 | 0.8 | 1 | 0.3 |
| 2nd | Pan de Azúcar | 9 | 1.9 | 3 | 3 |
| 2nd | Pan de Azúcar | 9 | 0.5 | 0.9 | 0.2 |
| 2nd | Pan de Azúcar | 9 | 1.2 | 1.8 | 1 |
| 2nd | Pan de Azúcar | 9 | 1.2 | 0.4 | 0.4 |
| 2nd | Pan de Azúcar | 9 | 2 | 5 | 2.5 |
| 2nd | Pan de Azúcar | 9 | 0.9 | 1.3 | 0.1 |
| 2nd | Pan de Azúcar | 9 | 1 | 2 | 0.2 |
| 2nd | Pan de Azúcar | 9 | 1.1 | 1.3 | 0.8 |
| 2nd | Pan de Azúcar | 9 | 1.2 | 2.6 | 1.1 |
| 2nd | Pan de Azúcar | 9 | 0.6 | 0.4 | 0.3 |
| 2nd | Pan de Azúcar | 10 | 0.6 | 1 | 0.2 |
| 2nd | Pan de Azúcar | 10 | 0.7 | 0.8 | 0.2 |
| 2nd | Pan de Azúcar | 10 | 0.6 | 0.7 | 0.2 |
| 2nd | Pan de Azúcar | 10 | 0.7 | 1 | 0.2 |
| 2nd | Pan de Azúcar | 10 | 0.9 | 1 | 0.3 |
| 2nd | Pan de Azúcar | 10 | 0.7 | 1.5 | 0.6 |
| 2nd | Pan de Azúcar | 10 | 2.5 | 3 | 2.5 |
| 2nd | Pan de Azúcar | 10 | 0.6 | 1.4 | 0.5 |
| 2nd | Pan de Azúcar | 10 | 2 | 1.8 | 1.2 |
| 2nd | Pan de Azúcar | 10 | 1.4 | 3 | 0.7 |
| 2nd | Pan de Azúcar | 10 | 1.5 | 3.5 | 1.2 |
| 2nd | Pan de Azúcar | 10 | 0.5 | 1 | 0.7 |
| 2nd | Pan de Azúcar | 10 | 2.9 | 3.1 | 2 |
| 2nd | Pan de Azúcar | 11 | 0.5 | 1.5 | 0.3 |
| 2nd | Pan de Azúcar | 11 | 0.9 | 0.6 | 0.6 |
| 2nd | Pan de Azúcar | 11 | 1 | 1.2 | 0.2 |
| 2nd | Pan de Azúcar | 11 | 0.9 | 1 | 0.3 |
| 2nd | Pan de Azúcar | 11 | 0.5 | 1.4 | 0.3 |
| 2nd | Pan de Azúcar | 11 | 0.8 | 1.2 | 0.5 |
| 2nd | Pan de Azúcar | 11 | 1.5 | 1.5 | 0.6 |
| 2nd | Pan de Azúcar | 11 | 1 | 1.3 | 1 |
| 2nd | Pan de Azúcar | 11 | 2.5 | 3 | 3 |
| 2nd | Pan de Azúcar | 11 | 1 | 1.1 | 0.5 |
| 2nd | Pan de Azúcar | 11 | 0.9 | 1.3 | 1 |
| 2nd | Pan de Azúcar | 11 | 1 | 1 | 0.2 |
| 2nd | Pan de Azúcar | 11 | 3.5 | 1.5 | 0.3 |
| 2nd | Pan de Azúcar | 11 | 1.6 | 2 | 1.2 |
| 2nd | Pan de Azúcar | 11 | 1 | 1 | 0.6 |
| 2nd | Pan de Azúcar | 11 | 0.9 | 1.4 | 1 |
| 2nd | Pan de Azúcar | 11 | 0.9 | 0.7 | 0.3 |
| 2nd | Pan de Azúcar | 12 | 1 | 1.5 | 0.2 |
| 2nd | Pan de Azúcar | 12 | 0.9 | 1.3 | 0.3 |
| 2nd | Pan de Azúcar | 12 | 1.2 | 2.5 | 1 |
| 2nd | Pan de Azúcar | 12 | 0.5 | 0.5 | 0.3 |
| 2nd | Pan de Azúcar | 12 | 1 | 0.9 | 0.5 |
| 2nd | Pan de Azúcar | 12 | 1 | 1.8 | 1.5 |
| 2nd | Pan de Azúcar | 12 | 0.6 | 1 | 0.3 |
| 2nd | Pan de Azúcar | 12 | 0.8 | 1.2 | 0.6 |
| 2nd | Pan de Azúcar | 12 | 1.5 | 1.3 | 0.8 |
| 2nd | Pan de Azúcar | 12 | 2.3 | 1.8 | 0.9 |
| 2nd | Pan de Azúcar | 12 | 1 | 0.6 | 0.5 |
| 2nd | Pan de Azúcar | 13 | 1 | 0.8 | 0.3 |
| 2nd | Pan de Azúcar | 13 | 0.5 | 1 | 0.4 |
| 2nd | Pan de Azúcar | 13 | 1 | 1.5 | 0.6 |
| 2nd | Pan de Azúcar | 13 | 2.5 | 4 | 1.5 |
| 2nd | Pan de Azúcar | 13 | 0.5 | 0.8 | 0.2 |
| 2nd | Pan de Azúcar | 13 | 1 | 1.5 | 0.6 |
| 2nd | Pan de Azúcar | 13 | 0.5 | 1 | 0.9 |
| 2nd | Pan de Azúcar | 13 | 1 | 0.8 | 0.6 |
| 2nd | Pan de Azúcar | 13 | 1 | 2 | 2 |
| 2nd | Pan de Azúcar | 13 | 2 | 3.5 | 2 |
| 2nd | Pan de Azúcar | 13 | 3 | 4 | 4 |
| 2nd | Pan de Azúcar | 13 | 3 | 3.5 | 5 |
| 2nd | Pan de Azúcar | 13 | 9 | 7.5 | 10 |
| 2nd | Pan de Azúcar | 13 | 1 | 1.5 | 1 |
| 2nd | Pan de Azúcar | 13 | 0.9 | 1 | 1 |
| 2nd | Pan de Azúcar | 13 | 3 | 3.5 | 4.5 |
| 2nd | Pan de Azúcar | 13 | 1 | 1.1 | 0.3 |
| 2nd | Pan de Azúcar | 13 | 1 | 0.6 | 0.5 |
| 2nd | Pan de Azúcar | 13 | 1 | 1.1 | 1.6 |
| 2nd | Pan de Azúcar | 13 | 1 | 0.6 | 0.9 |
| 2nd | Pan de Azúcar | 13 | 1 | 1.5 | 2 |
| 2nd | Pan de Azúcar | 14 | 0.6 | 0.5 | 0.3 |
| 2nd | Pan de Azúcar | 14 | 0.6 | 0.7 | 0.5 |
| 2nd | Pan de Azúcar | 14 | 0.5 | 1 | 0.9 |
| 2nd | Pan de Azúcar | 14 | 1 | 1 | 0.6 |
| 2nd | Pan de Azúcar | 14 | 0.7 | 0.9 | 0.2 |
| 2nd | Pan de Azúcar | 14 | 1 | 0.8 | 0.3 |
| 2nd | Pan de Azúcar | 14 | 2.5 | 4 | 2.5 |
| 2nd | Pan de Azúcar | 14 | 2.2 | 4.6 | 3.1 |
| 2nd | Pan de Azúcar | 14 | 1.2 | 1 | 1.3 |
| 2nd | Pan de Azúcar | 14 | 1 | 1 | 0.6 |
| 2nd | Pan de Azúcar | 14 | 1 | 1 | 0.2 |
| 2nd | Pan de Azúcar | 14 | 1.9 | 1.5 | 2.7 |
| 2nd | Pan de Azúcar | 14 | 3 | 4 | 3.2 |
| 2nd | Pan de Azúcar | 14 | 10.5 | 8.5 | 9.9 |
| 2nd | Pan de Azúcar | 14 | 1 | 0.6 | 0.2 |
| 2nd | Pan de Azúcar | 14 | 1 | 1 | 0.3 |
| 2nd | Pan de Azúcar | 14 | 0.5 | 0.9 | 0.3 |
| 2nd | Pan de Azúcar | 14 | 1 | 0.6 | 0.3 |
| 2nd | Pan de Azúcar | 14 | 0.7 | 0.8 | 0.8 |
| 2nd | Pan de Azúcar | 14 | 1 | 0.8 | 0.2 |
| 2nd | Pan de Azúcar | 14 | 0.7 | 0.6 | 0.2 |
| 2nd | Pan de Azúcar | 15 | 1.1 | 1 | 0.3 |
| 2nd | Pan de Azúcar | 15 | 0.8 | 0.9 | 0.2 |
| 2nd | Pan de Azúcar | 15 | 1 | 1.4 | 0.9 |
| 2nd | Pan de Azúcar | 15 | 0.7 | 0.9 | 0.3 |
| 2nd | Pan de Azúcar | 15 | 0.7 | 1 | 0.3 |
| 2nd | Pan de Azúcar | 15 | 0.9 | 0.9 | 0.4 |
| 2nd | Pan de Azúcar | 15 | 0.7 | 1.1 | 0.5 |
| 2nd | Pan de Azúcar | 15 | 1.3 | 1 | 0.5 |
| 2nd | Pan de Azúcar | 15 | 0.8 | 0.6 | 0.2 |
| 2nd | Pan de Azúcar | 15 | 0.7 | 0.5 | 0.1 |
| 2nd | Pan de Azúcar | 15 | 0.6 | 1 | 1.3 |
| 2nd | Pan de Azúcar | 15 | 0.8 | 1 | 0.1 |
| 2nd | Pan de Azúcar | 16 | 1 | 0.8 | 0.3 |
| 2nd | Pan de Azúcar | 16 | 3 | 2.4 | 3 |
| 2nd | Pan de Azúcar | 16 | 0.5 | 1 | 0.3 |
| 2nd | Pan de Azúcar | 16 | 1.1 | 1.9 | 0.7 |
| 2nd | Pan de Azúcar | 16 | 4.3 | 4.4 | 1.7 |
| 2nd | Pan de Azúcar | 16 | 2 | 2.7 | 2.1 |
| 2nd | Pan de Azúcar | 17 | 1 | 1 | 0.3 |
| 2nd | Pan de Azúcar | 17 | 1.5 | 1.5 | 0.4 |
| 2nd | Pan de Azúcar | 17 | 2 | 2.5 | 3.5 |
| 2nd | Pan de Azúcar | 17 | 0.9 | 0.9 | 0.3 |
| 2nd | Pan de Azúcar | 17 | 0.6 | 0.5 | 0.2 |
| 2nd | Pan de Azúcar | 17 | 1.5 | 1.1 | 1 |
| 2nd | Pan de Azúcar | 17 | 0.9 | 1.5 | 1.3 |
| 2nd | Pan de Azúcar | 17 | 0.7 | 0.9 | 0.5 |
| 2nd | Pan de Azúcar | 17 | 0.9 | 0.9 | 0.4 |
| 2nd | Pan de Azúcar | 17 | 1 | 1.3 | 0.6 |
| 2nd | Pan de Azúcar | 17 | 0.1 | 1.1 | 0.6 |
| 2nd | Pan de Azúcar | 17 | 0.5 | 1 | 0.3 |
| 2nd | Pan de Azúcar | 17 | 0.7 | 0.8 | 0.3 |
| 2nd | Pan de Azúcar | 17 | 0.9 | 1.2 | 0.8 |
| 2nd | Pan de Azúcar | 17 | 0.8 | 0.8 | 0.2 |
| 2nd | Pan de Azúcar | 17 | 0.4 | 1 | 0.4 |
| 2nd | Pan de Azúcar | 17 | 1 | 0.8 | 0.3 |
| 2nd | Pan de Azúcar | 17 | 0.6 | 1 | 0.5 |
| 2nd | Pan de Azúcar | 17 | 0.8 | 0.9 | 0.2 |
| 2nd | Pan de Azúcar | 17 | 1.2 | 2 | 0.3 |
| 2nd | Pan de Azúcar | 17 | 0.9 | 0.9 | 0.3 |
| 2nd | Pan de Azúcar | 17 | 0.6 | 1.2 | 0.5 |
| 2nd | Pan de Azúcar | 17 | 1 | 1.3 | 0.8 |
| 2nd | Pan de Azúcar | 17 | 1.5 | 2 | 1 |
| 2nd | Pan de Azúcar | 17 | 1 | 1 | 0.3 |
| 2nd | Pan de Azúcar | 17 | 0.6 | 1 | 0.6 |
| 2nd | Pan de Azúcar | 18 | 1.5 | 0.9 | 0.6 |
| 2nd | Pan de Azúcar | 18 | 2.5 | 3 | 1 |
| 2nd | Pan de Azúcar | 18 | 0.8 | 0.7 | 0.8 |
| 2nd | Pan de Azúcar | 18 | 1 | 0.7 | 0.4 |
| 2nd | Pan de Azúcar | 18 | 0.6 | 0.5 | 0.3 |
| 2nd | Pan de Azúcar | 18 | 3 | 2 | 1.5 |
| 2nd | Pan de Azúcar | 18 | 1.3 | 2 | 2 |
| 2nd | Pan de Azúcar | 18 | 5.5 | 9 | 6 |
| 2nd | Pan de Azúcar | 18 | 0.9 | 1.5 | 0.7 |
| 2nd | Pan de Azúcar | 18 | 1 | 1.3 | 1.6 |
| 2nd | Pan de Azúcar | 18 | 0.8 | 1.4 | 0.9 |
| 2nd | Pan de Azúcar | 18 | 4 | 3 | 4 |
| 2nd | Pan de Azúcar | 18 | 2.5 | 2.5 | 1.5 |
| 2nd | Pan de Azúcar | 18 | 8.5 | 5 | 8.5 |
| 2nd | Pan de Azúcar | 18 | 3.5 | 3.5 | 3 |
| 2nd | Pan de Azúcar | 18 | 1 | 1.5 | 1 |
| 2nd | Pan de Azúcar | 18 | 1 | 1.3 | 0.3 |
| 2nd | Pan de Azúcar | 18 | 2.5 | 2 | 2.7 |
| 2nd | Pan de Azúcar | 18 | 5 | 7.5 | 6.5 |
| 2nd | Pan de Azúcar | 18 | 0.7 | 0.5 | 0.4 |
| 2nd | Pan de Azúcar | 18 | 0.5 | 0.6 | 0.3 |
| 2nd | Pan de Azúcar | 18 | 0.9 | 0.8 | 0.3 |
| 2nd | Pan de Azúcar | 18 | 3 | 3 | 3.2 |
| 2nd | Pan de Azúcar | 18 | 0.9 | 1 | 0.5 |
| 2nd | Pan de Azúcar | 18 | 0.7 | 0.4 | 0.3 |
| 2nd | Pan de Azúcar | 18 | 0.9 | 1 | 0.6 |
| 2nd | Pan de Azúcar | 18 | 0.6 | 0.7 | 0.3 |
| 2nd | Pan de Azúcar | 18 | 0.8 | 1.5 | 0.8 |
| 2nd | Pan de Azúcar | 18 | 0.7 | 1 | 0.6 |
| 2nd | Pan de Azúcar | 19 | 1 | 1 | 0.5 |
| 2nd | Pan de Azúcar | 19 | 1.2 | 1 | 0.4 |
| 2nd | Pan de Azúcar | 19 | 0.8 | 2 | 0.5 |
| 2nd | Pan de Azúcar | 19 | 0.6 | 1 | 0.3 |
| 2nd | Pan de Azúcar | 19 | 0.7 | 1 | 0.4 |
| 2nd | Pan de Azúcar | 19 | 0.5 | 0.9 | 0.3 |
| 2nd | Pan de Azúcar | 19 | 1.5 | 1 | 0.7 |
| 2nd | Pan de Azúcar | 19 | 2 | 2.2 | 0.8 |
| 2nd | Pan de Azúcar | 19 | 0.9 | 1.4 | 1 |
| 2nd | Pan de Azúcar | 19 | 0.8 | 0.8 | 0.3 |
| 2nd | Pan de Azúcar | 19 | 0.3 | 0.5 | 0.1 |
| 2nd | Pan de Azúcar | 19 | 0.8 | 1 | 0.4 |
| 2nd | Pan de Azúcar | 19 | 0.9 | 1 | 0.4 |
| 2nd | Pan de Azúcar | 19 | 1 | 0.9 | 0.5 |
| 2nd | Pan de Azúcar | 20 | 0.9 | 1 | 0.5 |
| 2nd | Pan de Azúcar | 20 | 0.4 | 0.4 | 0.2 |
| 2nd | Pan de Azúcar | 20 | 0.6 | 0.7 | 0.3 |
| 2nd | Pan de Azúcar | 20 | 0.7 | 0.7 | 0.6 |
| 2nd | Pan de Azúcar | 20 | 2 | 1.5 | 0.8 |
| 2nd | Pan de Azúcar | 20 | 1 | 2 | 1.3 |
| 2nd | Pan de Azúcar | 20 | 0.9 | 1 | 0.6 |
| 2nd | Pan de Azúcar | 20 | 0.7 | 0.8 | 0.4 |
| 2nd | Pan de Azúcar | 20 | 0.7 | 0.5 | 0.2 |
| 2nd | Pan de Azúcar | 20 | 1 | 1 | 0.9 |
| 2nd | Pan de Azúcar | 20 | 1 | 1.1 | 0.4 |
| 2nd | Pan de Azúcar | 20 | 1 | 0.9 | 0.3 |
| 2nd | Pan de Azúcar | 20 | 1 | 1 | 0.6 |
| 2nd | Pan de Azúcar | 20 | 0.7 | 1.5 | 0.7 |
| 2nd | Pan de Azúcar | 20 | 0.7 | 0.6 | 0.4 |
| 2nd | Pan de Azúcar | 20 | 0.5 | 0.7 | 0.1 |
| 2nd | Pan de Azúcar | 20 | 0.7 | 0.9 | 0.3 |
| 2nd | Pan de Azúcar | 20 | 0.5 | 0.7 | 0.6 |
| 2nd | Pan de Azúcar | 20 | 1.2 | 1.7 | 0.9 |
| 2nd | Pan de Azúcar | 20 | 0.9 | 1.1 | 0.3 |
| 2nd | Santa Gracia | 1 | 8.5 | 7 | 6 |
| 2nd | Santa Gracia | 1 | 3 | 2 | 2.5 |
| 2nd | Santa Gracia | 1 | 4 | 4.5 | 4.5 |
| 2nd | Santa Gracia | 1 | 5.5 | 5.5 | 6.5 |
| 2nd | Santa Gracia | 1 | 2.5 | 5 | 6 |
| 2nd | Santa Gracia | 1 | 7 | 6 | 9 |
| 2nd | Santa Gracia | 1 | 4 | 3 | 5 |
| 2nd | Santa Gracia | 1 | 4 | 7 | 4 |
| 2nd | Santa Gracia | 1 | 7 | 10 | 3 |
| 2nd | Santa Gracia | 1 | 5.5 | 6.5 | 7 |
| 2nd | Santa Gracia | 1 | 7 | 8 | 9 |
| 2nd | Santa Gracia | 1 | 4.5 | 10 | 5 |
| 2nd | Santa Gracia | 1 | 6 | 7 | 9 |
| 2nd | Santa Gracia | 1 | 6 | 10 | 3 |
| 2nd | Santa Gracia | 1 | 5 | 9 | 6 |
| 2nd | Santa Gracia | 1 | 2.5 | 4 | 4.5 |
| 2nd | Santa Gracia | 1 | 7 | 9 | 5 |
| 2nd | Santa Gracia | 1 | 5 | 9 | 6 |
| 2nd | Santa Gracia | 1 | 5 | 10 | 8 |
| 2nd | Santa Gracia | 1 | 8 | 8.5 | 8 |
| 2nd | Santa Gracia | 1 | 9 | 8 | 5 |
| 2nd | Santa Gracia | 1 | 6 | 9 | 4 |
| 2nd | Santa Gracia | 1 | 5.5 | 5.5 | 6 |
| 2nd | Santa Gracia | 1 | 13 | 8 | 7 |
| 2nd | Santa Gracia | 1 | 11 | 8.5 | 7 |
| 2nd | Santa Gracia | 1 | 4.5 | 5.5 | 6 |
| 2nd | Santa Gracia | 1 | 7 | 8 | 5 |
| 2nd | Santa Gracia | 1 | 5 | 9 | 1.5 |
| 2nd | Santa Gracia | 1 | 3 | 2.5 | 3.5 |
| 2nd | Santa Gracia | 1 | 4 | 4.5 | 4.5 |
| 2nd | Santa Gracia | 1 | 4 | 8 | 4 |
| 2nd | Santa Gracia | 1 | 4.5 | 7 | 5 |
| 2nd | Santa Gracia | 1 | 5 | 5 | 8 |
| 2nd | Santa Gracia | 1 | 4.5 | 7 | 6 |
| 2nd | Santa Gracia | 1 | 6 | 7 | 5.5 |
| 2nd | Santa Gracia | 1 | 3 | 6 | 7.5 |
| 2nd | Santa Gracia | 1 | 3 | 6 | 2.5 |
| 2nd | Santa Gracia | 2 | 2 | 2.4 | 1.9 |
| 2nd | Santa Gracia | 2 | 0.6 | 0.7 | 0.5 |
| 2nd | Santa Gracia | 2 | 0.6 | 0.4 | 0.3 |
| 2nd | Santa Gracia | 2 | 2.1 | 1 | 1 |
| 2nd | Santa Gracia | 2 | 1 | 0.9 | 0.7 |
| 2nd | Santa Gracia | 2 | 1 | 0.6 | 0.5 |
| 2nd | Santa Gracia | 2 | 1.6 | 1.5 | 0.9 |
| 2nd | Santa Gracia | 2 | 2 | 0.9 | 1 |
| 2nd | Santa Gracia | 2 | 1.3 | 1.2 | 1.9 |
| 2nd | Santa Gracia | 2 | 0.7 | 1.2 | 1 |
| 2nd | Santa Gracia | 2 | 0.9 | 1.3 | 1.4 |
| 2nd | Santa Gracia | 2 | 1.1 | 1.5 | 1 |
| 2nd | Santa Gracia | 2 | 3 | 3.5 | 2.3 |
| 2nd | Santa Gracia | 2 | 1.4 | 1.2 | 1 |
| 2nd | Santa Gracia | 2 | 1 | 2.4 | 0.6 |
| 2nd | Santa Gracia | 2 | 2.4 | 5 | 3.4 |
| 2nd | Santa Gracia | 2 | 1.1 | 1.3 | 1.9 |
| 2nd | Santa Gracia | 2 | 1.4 | 1 | 0.4 |
| 2nd | Santa Gracia | 2 | 2.5 | 3.3 | 2.4 |
| 2nd | Santa Gracia | 2 | 1.5 | 1.2 | 0.7 |
| 2nd | Santa Gracia | 2 | 0.9 | 0.9 | 0.6 |
| 2nd | Santa Gracia | 2 | 1.1 | 0.9 | 1 |
| 2nd | Santa Gracia | 2 | 1.5 | 1 | 0.7 |
| 2nd | Santa Gracia | 2 | 1.6 | 2.8 | 0.6 |
| 2nd | Santa Gracia | 2 | 1.6 | 1.9 | 1 |
| 2nd | Santa Gracia | 2 | 1.1 | 1 | 0.7 |
| 2nd | Santa Gracia | 2 | 2.4 | 2.7 | 2.3 |
| 2nd | Santa Gracia | 2 | 2 | 1.8 | 1.9 |
| 2nd | Santa Gracia | 2 | 1.3 | 1.7 | 2.2 |
| 2nd | Santa Gracia | 2 | 2.2 | 1.3 | 1.5 |
| 2nd | Santa Gracia | 2 | 1.2 | 1.4 | 1 |
| 2nd | Santa Gracia | 3 | 3.5 | 3 | 2.2 |
| 2nd | Santa Gracia | 3 | 3.1 | 2.7 | 3 |
| 2nd | Santa Gracia | 3 | 5 | 4.5 | 5.7 |
| 2nd | Santa Gracia | 3 | 4 | 5 | 2.7 |
| 2nd | Santa Gracia | 3 | 6.7 | 5.2 | 2 |
| 2nd | Santa Gracia | 3 | 6.1 | 9 | 5.1 |
| 2nd | Santa Gracia | 3 | 3.6 | 2.4 | 1.3 |
| 2nd | Santa Gracia | 3 | 3 | 2.5 | 1.6 |
| 2nd | Santa Gracia | 3 | 6.4 | 7 | 2.8 |
| 2nd | Santa Gracia | 3 | 5.3 | 4.7 | 2.9 |
| 2nd | Santa Gracia | 3 | 3.2 | 4.6 | 1 |
| 2nd | Santa Gracia | 3 | 1.5 | 2.2 | 0.8 |
| 2nd | Santa Gracia | 3 | 3.7 | 3.2 | 1.6 |
| 2nd | Santa Gracia | 3 | 2.7 | 2.6 | 2 |
| 2nd | Santa Gracia | 3 | 3.1 | 2.8 | 1.6 |
| 2nd | Santa Gracia | 3 | 6 | 0.8 | 0.9 |
| 2nd | Santa Gracia | 3 | 4 | 4.9 | 2.6 |
| 2nd | Santa Gracia | 3 | 2.6 | 2.7 | 1.7 |
| 2nd | Santa Gracia | 3 | 2.1 | 3.7 | 1.9 |
| 2nd | Santa Gracia | 4 | 2 | 3.5 | 1.7 |
| 2nd | Santa Gracia | 4 | 2 | 1.8 | 1.5 |
| 2nd | Santa Gracia | 4 | 10 | 4.5 | 4 |
| 2nd | Santa Gracia | 4 | 8 | 7.5 | 3 |
| 2nd | Santa Gracia | 4 | 1.5 | 2.2 | 0.9 |
| 2nd | Santa Gracia | 4 | 3 | 2.5 | 1.5 |
| 2nd | Santa Gracia | 4 | 1.5 | 2 | 1.7 |
| 2nd | Santa Gracia | 4 | 6 | 6.5 | 3.4 |
| 2nd | Santa Gracia | 4 | 2.5 | 2 | 1.7 |
| 2nd | Santa Gracia | 4 | 3.5 | 2.2 | 2.2 |
| 2nd | Santa Gracia | 4 | 2.5 | 2 | 1.2 |
| 2nd | Santa Gracia | 4 | 1.5 | 2 | 1 |
| 2nd | Santa Gracia | 4 | 1.3 | 1 | 1.3 |
| 2nd | Santa Gracia | 4 | 3.4 | 3 | 2.9 |
| 2nd | Santa Gracia | 4 | 1.1 | 1.2 | 0.8 |
| 2nd | Santa Gracia | 4 | 1.2 | 1.2 | 1.9 |
| 2nd | Santa Gracia | 4 | 15 | 12 | 11 |
| 2nd | Santa Gracia | 4 | 1.2 | 1.8 | 2.2 |
| 2nd | Santa Gracia | 4 | 0.7 | 0.6 | 0.6 |
| 2nd | Santa Gracia | 4 | 1 | 1.5 | 0.6 |
| 2nd | Santa Gracia | 4 | 2.3 | 4.6 | 1.2 |
| 2nd | Santa Gracia | 4 | 0.8 | 1.2 | 1 |
| 2nd | Santa Gracia | 4 | 1.5 | 1.2 | 0.7 |
| 2nd | Santa Gracia | 4 | 1.7 | 1.4 | 1.2 |
| 2nd | Santa Gracia | 5 | 8 | 7 | 6 |
| 2nd | Santa Gracia | 5 | 4 | 5 | 8.5 |
| 2nd | Santa Gracia | 5 | 4 | 5.5 | 2.5 |
| 2nd | Santa Gracia | 5 | 4 | 7 | 6.5 |
| 2nd | Santa Gracia | 5 | 5.5 | 7.5 | 4.7 |
| 2nd | Santa Gracia | 5 | 3 | 4.5 | 2 |
| 2nd | Santa Gracia | 5 | 5 | 7.3 | 4.3 |
| 2nd | Santa Gracia | 5 | 9.5 | 7.6 | 6.5 |
| 2nd | Santa Gracia | 5 | 5 | 4.5 | 10 |
| 2nd | Santa Gracia | 5 | 8 | 8 | 6 |
| 2nd | Santa Gracia | 5 | 4 | 5.3 | 6.2 |
| 2nd | Santa Gracia | 5 | 4.5 | 2.5 | 5 |
| 2nd | Santa Gracia | 5 | 1.5 | 2 | 1.7 |
| 2nd | Santa Gracia | 5 | 4.5 | 7.5 | 2.6 |
| 2nd | Santa Gracia | 5 | 9.5 | 7 | 8.5 |
| 2nd | Santa Gracia | 5 | 1 | 2.5 | 0.8 |
| 2nd | Santa Gracia | 6 | 8 | 10 | 6 |
| 2nd | Santa Gracia | 6 | 4 | 5 | 2.5 |
| 2nd | Santa Gracia | 6 | 3 | 6 | 2 |
| 2nd | Santa Gracia | 6 | 6.5 | 11 | 5 |
| 2nd | Santa Gracia | 6 | 3 | 4 | 3 |
| 2nd | Santa Gracia | 6 | 7.5 | 6 | 4.5 |
| 2nd | Santa Gracia | 6 | 6 | 5 | 4 |
| 2nd | Santa Gracia | 6 | 3 | 6.5 | 9 |
| 2nd | Santa Gracia | 6 | 10 | 8.5 | 8 |
| 2nd | Santa Gracia | 6 | 4.5 | 7 | 5 |
| 2nd | Santa Gracia | 6 | 4.5 | 4 | 5 |
| 2nd | Santa Gracia | 6 | 4.5 | 6.5 | 3 |
| 2nd | Santa Gracia | 6 | 8 | 9 | 7 |
| 2nd | Santa Gracia | 6 | 10 | 11 | 8 |
| 2nd | Santa Gracia | 6 | 5.5 | 8 | 4 |
| 2nd | Santa Gracia | 6 | 3.5 | 3 | 2.9 |
| 2nd | Santa Gracia | 6 | 2.5 | 5 | 1.5 |
| 2nd | Santa Gracia | 6 | 7 | 8 | 6 |
| 2nd | Santa Gracia | 6 | 5 | 8 | 4 |
| 2nd | Santa Gracia | 6 | 4.5 | 4 | 3.5 |
| 2nd | Santa Gracia | 6 | 4 | 6 | 3.5 |
| 2nd | Santa Gracia | 6 | 5.5 | 5.5 | 3 |
| 2nd | Santa Gracia | 6 | 5 | 7.5 | 5.5 |
| 2nd | Santa Gracia | 6 | 4 | 7 | 5 |
| 2nd | Santa Gracia | 6 | 3 | 6 | 6.5 |
| 2nd | Santa Gracia | 6 | 2 | 4 | 2.5 |
| 2nd | Santa Gracia | 6 | 6 | 5.5 | 5 |
| 2nd | Santa Gracia | 6 | 5.5 | 6.5 | 3 |
| 2nd | Santa Gracia | 6 | 9.5 | 9 | 3 |
| 2nd | Santa Gracia | 6 | 4 | 4.5 | 10 |
| 2nd | Santa Gracia | 6 | 5 | 5.5 | 18 |
| 2nd | Santa Gracia | 7 | 0.5 | 0.5 | 0.3 |
| 2nd | Santa Gracia | 7 | 7.5 | 6 | 8 |
| 2nd | Santa Gracia | 7 | 4 | 4 | 3 |
| 2nd | Santa Gracia | 7 | 1.5 | 1.2 | 1.4 |
| 2nd | Santa Gracia | 7 | 2 | 2 | 1.5 |
| 2nd | Santa Gracia | 7 | 1.7 | 1.5 | 1.6 |
| 2nd | Santa Gracia | 8 | 1.4 | 1 | 0.8 |
| 2nd | Santa Gracia | 8 | 0.9 | 0.9 | 0.3 |
| 2nd | Santa Gracia | 8 | 1 | 2.3 | 0.4 |
| 2nd | Santa Gracia | 8 | 2.7 | 3 | 1.2 |
| 2nd | Santa Gracia | 8 | 1.2 | 0.8 | 0.6 |
| 2nd | Santa Gracia | 8 | 1.9 | 1.4 | 0.7 |
| 2nd | Santa Gracia | 8 | 1.5 | 1.4 | 1.1 |
| 2nd | Santa Gracia | 8 | 1 | 1 | 0.6 |
| 2nd | Santa Gracia | 8 | 1.4 | 1.3 | 0.7 |
| 2nd | Santa Gracia | 8 | 1.5 | 3 | 1.3 |
| 2nd | Santa Gracia | 8 | 1.5 | 1.9 | 1.6 |
| 2nd | Santa Gracia | 8 | 1.5 | 2 | 0.5 |
| 2nd | Santa Gracia | 8 | 2 | 2.5 | 2.6 |
| 2nd | Santa Gracia | 8 | 2 | 2.3 | 2.1 |
| 2nd | Santa Gracia | 8 | 1 | 1.5 | 0.3 |
| 2nd | Santa Gracia | 9 | 1.5 | 3 | 1.5 |
| 2nd | Santa Gracia | 9 | 1 | 1.5 | 1.5 |
| 2nd | Santa Gracia | 9 | 1.4 | 1.5 | 2.6 |
| 2nd | Santa Gracia | 9 | 1.4 | 1.8 | 2.7 |
| 2nd | Santa Gracia | 9 | 1.5 | 2.3 | 1 |
| 2nd | Santa Gracia | 9 | 1.4 | 1.6 | 0.8 |
| 2nd | Santa Gracia | 9 | 0.9 | 1.4 | 1 |
| 2nd | Santa Gracia | 9 | 1.2 | 1.2 | 1.8 |
| 2nd | Santa Gracia | 9 | 2.5 | 1 | 7.9 |
| 2nd | Santa Gracia | 10 | 1.8 | 3 | 0.4 |
| 2nd | Santa Gracia | 10 | 3.5 | 5.5 | 2.4 |
| 2nd | Santa Gracia | 10 | 4 | 4.5 | 1.7 |
| 2nd | Santa Gracia | 10 | 1.2 | 2 | 0.5 |
| 2nd | Santa Gracia | 10 | 4.5 | 6.7 | 8 |
| 2nd | Santa Gracia | 10 | 2 | 3.5 | 1 |
| 2nd | Santa Gracia | 10 | 4.7 | 5.1 | 4.3 |
| 2nd | Santa Gracia | 10 | 2.4 | 3.1 | 1.2 |
| 2nd | Santa Gracia | 10 | 1 | 1.4 | 0.6 |
| 2nd | Santa Gracia | 10 | 1.5 | 1.4 | 0.9 |
| 2nd | Santa Gracia | 10 | 1.5 | 1.7 | 1.8 |
| 2nd | Santa Gracia | 11 | 0.6 | 0.5 | 0.5 |
| 2nd | Santa Gracia | 11 | 1.7 | 1.3 | 0.8 |
| 2nd | Santa Gracia | 11 | 0.9 | 0.8 | 0.8 |
| 2nd | Santa Gracia | 11 | 1.5 | 1.7 | 1.7 |
| 2nd | Santa Gracia | 11 | 1.9 | 1.4 | 1.1 |
| 2nd | Santa Gracia | 11 | 1.9 | 2 | 1.7 |
| 2nd | Santa Gracia | 11 | 2.3 | 2.2 | 1.8 |
| 2nd | Santa Gracia | 11 | 1.7 | 2.3 | 2 |
| 2nd | Santa Gracia | 11 | 3 | 6.1 | 2.4 |
| 2nd | Santa Gracia | 11 | 1.9 | 1.9 | 2 |
| 2nd | Santa Gracia | 11 | 2 | 3.4 | 1 |
| 2nd | Santa Gracia | 11 | 1 | 1.4 | 1.2 |
| 2nd | Santa Gracia | 11 | 1 | 1.5 | 1.3 |
| 2nd | Santa Gracia | 11 | 1.4 | 2 | 3.4 |
| 2nd | Santa Gracia | 11 | 0.7 | 0.7 | 0.4 |
| 2nd | Santa Gracia | 11 | 1.5 | 1.7 | 1.6 |
| 2nd | Santa Gracia | 11 | 5 | 9.3 | 5 |
| 2nd | Santa Gracia | 12 | 1.7 | 2 | 0.9 |
| 2nd | Santa Gracia | 12 | 0.9 | 1 | 0.5 |
| 2nd | Santa Gracia | 12 | 1.4 | 1.3 | 1.5 |
| 2nd | Santa Gracia | 12 | 1 | 0.9 | 0.6 |
| 2nd | Santa Gracia | 12 | 0.5 | 0.4 | 0.2 |
| 2nd | Santa Gracia | 12 | 1.4 | 0.9 | 0.3 |
| 2nd | Santa Gracia | 12 | 2.5 | 0.5 | 4.5 |
| 2nd | Santa Gracia | 12 | 2 | 1.1 | 1.8 |
| 2nd | Santa Gracia | 12 | 1.2 | 1 | 0.5 |
| 2nd | Santa Gracia | 12 | 1.5 | 3 | 1.3 |
| 2nd | Santa Gracia | 12 | 1.7 | 2.8 | 1.5 |
| 2nd | Santa Gracia | 12 | 2.4 | 1.5 | 0.6 |
| 2nd | Santa Gracia | 12 | 3.1 | 1.9 | 2 |
| 2nd | Santa Gracia | 12 | 1.4 | 2.6 | 0.9 |
| 2nd | Santa Gracia | 12 | 1.4 | 1.4 | 1.2 |
| 2nd | Santa Gracia | 12 | 1.6 | 2 | 1.1 |
| 2nd | Santa Gracia | 12 | 0.8 | 0.9 | 0.7 |
| 2nd | Santa Gracia | 12 | 1 | 2 | 1.2 |
| 2nd | Santa Gracia | 13 | 4 | 5 | 5 |
| 2nd | Santa Gracia | 13 | 6.5 | 8 | 5.5 |
| 2nd | Santa Gracia | 13 | 3 | 1.8 | 1.5 |
| 2nd | Santa Gracia | 13 | 3 | 3 | 1.2 |
| 2nd | Santa Gracia | 13 | 5 | 7 | 4.5 |
| 2nd | Santa Gracia | 13 | 1.5 | 1.2 | 1.5 |
| 2nd | Santa Gracia | 13 | 1.1 | 0.9 | 0.2 |
| 2nd | Santa Gracia | 13 | 2 | 2.5 | 4.5 |
| 2nd | Santa Gracia | 13 | 1 | 1.3 | 0.9 |
| 2nd | Santa Gracia | 13 | 0.8 | 1 | 0.7 |
| 2nd | Santa Gracia | 13 | 6.2 | 7.5 | 5.7 |
| 2nd | Santa Gracia | 14 | 2.2 | 1.5 | 0.8 |
| 2nd | Santa Gracia | 14 | 1.5 | 3 | 1 |
| 2nd | Santa Gracia | 14 | 1.5 | 1.5 | 0.9 |
| 2nd | Santa Gracia | 14 | 2 | 1 | 0.6 |
| 2nd | Santa Gracia | 14 | 1.4 | 1.9 | 1.2 |
| 2nd | Santa Gracia | 14 | 3 | 2.5 | 2.4 |
| 2nd | Santa Gracia | 15 | 2 | 2.5 | 1.3 |
| 2nd | Santa Gracia | 15 | 0.5 | 0.5 | 0.2 |
| 2nd | Santa Gracia | 15 | 0.7 | 0.6 | 0.4 |
| 2nd | Santa Gracia | 15 | 2 | 1.7 | 1.7 |
| 2nd | Santa Gracia | 15 | 1.5 | 2.1 | 1.8 |
| 2nd | Santa Gracia | 15 | 1 | 0.9 | 0.3 |
| 2nd | Santa Gracia | 15 | 1 | 1.5 | 0.9 |
| 2nd | Santa Gracia | 16 | 0.8 | 0.6 | 0.5 |
| 2nd | Santa Gracia | 16 | 1.1 | 1.4 | 1.8 |
| 2nd | Santa Gracia | 16 | 1 | 1.2 | 1.7 |
| 2nd | Santa Gracia | 16 | 0.8 | 0.8 | 0.5 |
| 2nd | Santa Gracia | 16 | 3 | 2.8 | 1.4 |
| 2nd | Santa Gracia | 16 | 3.5 | 2.5 | 4.5 |
| 2nd | Santa Gracia | 16 | 1.5 | 2 | 3 |
| 2nd | Santa Gracia | 16 | 2 | 3.1 | 4.5 |
| 2nd | Santa Gracia | 16 | 1.7 | 1 | 1.1 |
| 2nd | Santa Gracia | 16 | 1.4 | 1.5 | 1.2 |
| 2nd | Santa Gracia | 16 | 3.1 | 3.2 | 3.4 |
| 2nd | Santa Gracia | 16 | 5.5 | 4 | 3.8 |
| 2nd | Santa Gracia | 16 | 2.5 | 3.5 | 1 |
| 2nd | Santa Gracia | 16 | 1.9 | 1.5 | 0.6 |
| 2nd | Santa Gracia | 16 | 1 | 2.5 | 0.9 |
| 2nd | Santa Gracia | 17 | 1 | 0.9 | 0.7 |
| 2nd | Santa Gracia | 17 | 3 | 1.8 | 1.2 |
| 2nd | Santa Gracia | 17 | 1.3 | 1.7 | 0.6 |
| 2nd | Santa Gracia | 17 | 1.1 | 1 | 1.2 |
| 2nd | Santa Gracia | 17 | 1 | 2.9 | 0.7 |
| 2nd | Santa Gracia | 17 | 1.3 | 0.9 | 1.7 |
| 2nd | Santa Gracia | 17 | 0.9 | 1.5 | 1.2 |
| 2nd | Santa Gracia | 17 | 1.8 | 1.9 | 1.2 |
| 2nd | Santa Gracia | 17 | 1.4 | 2 | 0.6 |
| 2nd | Santa Gracia | 17 | 0.9 | 1 | 0.3 |
| 2nd | Santa Gracia | 17 | 0.8 | 1.1 | 0.9 |
| 2nd | Santa Gracia | 17 | 2 | 1.8 | 1.5 |
| 2nd | Santa Gracia | 17 | 1.9 | 1.6 | 1.4 |
| 2nd | Santa Gracia | 17 | 1.8 | 2.2 | 0.9 |
| 2nd | Santa Gracia | 17 | 1 | 1 | 0.5 |
| 2nd | Santa Gracia | 17 | 1.7 | 2.1 | 2.1 |
| 2nd | Santa Gracia | 18 | 2.1 | 2.2 | 1.4 |
| 2nd | Santa Gracia | 18 | 5.5 | 6 | 3.9 |
| 2nd | Santa Gracia | 18 | 4.7 | 6.1 | 2.4 |
| 2nd | Santa Gracia | 18 | 1.1 | 2.4 | 0.8 |
| 2nd | Santa Gracia | 18 | 1.2 | 2.3 | 1 |
| 2nd | Santa Gracia | 18 | 2.8 | 2.9 | 3.2 |
| 2nd | Santa Gracia | 18 | 6 | 7.1 | 2.7 |
| 2nd | Santa Gracia | 18 | 2 | 1.7 | 2.2 |
| 2nd | Santa Gracia | 18 | 2.1 | 2.5 | 1.7 |
| 2nd | Santa Gracia | 18 | 4.5 | 5 | 1.9 |
| 2nd | Santa Gracia | 18 | 1.6 | 1.4 | 0.8 |
| 2nd | Santa Gracia | 18 | 1.5 | 1.3 | 0.4 |
| 2nd | Santa Gracia | 18 | 2.6 | 2.7 | 2.3 |
| 2nd | Santa Gracia | 18 | 3.5 | 2.5 | 1.4 |
| 2nd | Santa Gracia | 18 | 3.7 | 4.6 | 1.9 |
| 2nd | Santa Gracia | 18 | 1.8 | 1.7 | 1.6 |
| 2nd | Santa Gracia | 18 | 2 | 2.8 | 1.8 |
| 2nd | Santa Gracia | 18 | 1.4 | 2.9 | 0.7 |
| 2nd | Santa Gracia | 18 | 2 | 1.8 | 0.8 |
| 2nd | Santa Gracia | 19 | 2.9 | 2.4 | 1.1 |
| 2nd | Santa Gracia | 19 | 1 | 2.5 | 0.9 |
| 2nd | Santa Gracia | 19 | 6 | 7.4 | 4 |
| 2nd | Santa Gracia | 19 | 2.2 | 2.9 | 1.8 |
| 2nd | Santa Gracia | 19 | 1.3 | 1.4 | 0.4 |
| 2nd | Santa Gracia | 19 | 6.5 | 7 | 2.7 |
| 2nd | Santa Gracia | 19 | 1.3 | 2.9 | 0.6 |
| 2nd | Santa Gracia | 19 | 4 | 6.5 | 5 |
| 2nd | Santa Gracia | 19 | 3.7 | 6.4 | 3.1 |
| 2nd | Santa Gracia | 19 | 0.9 | 0.7 | 0.5 |
| 2nd | Santa Gracia | 19 | 1.7 | 3 | 1 |
| 2nd | Santa Gracia | 19 | 1.9 | 3.2 | 1.9 |
| 2nd | Santa Gracia | 20 | 1 | 1.5 | 1 |
| 2nd | Santa Gracia | 20 | 3.5 | 1.5 | 1.9 |
| 2nd | Santa Gracia | 20 | 2.4 | 1.9 | 0.4 |
| 2nd | Santa Gracia | 20 | 0.6 | 0.7 | 0.3 |
| 2nd | Santa Gracia | 20 | 0.8 | 1.8 | 0.7 |
| 2nd | Santa Gracia | 20 | 0.6 | 0.6 | 0.5 |
| 2nd | Santa Gracia | 20 | 0.8 | 1 | 0.3 |
| 2nd | Santa Gracia | 20 | 1 | 1.2 | 0.5 |
| 2nd | Santa Gracia | 20 | 1.8 | 1.4 | 1.8 |
| 2nd | Santa Gracia | 20 | 1 | 2.8 | 0.4 |
| 2nd | Santa Gracia | 20 | 0.9 | 2.5 | 1.2 |
| 2nd | Santa Gracia | 20 | 0.4 | 0.5 | 0.6 |
| 2nd | Santa Gracia | 20 | 1.4 | 2 | 0.6 |
| 2nd | La CampaNahuelbuta | 1 | 2.5 | 3 | 0.6 |
| 2nd | La CampaNahuelbuta | 1 | 0.7 | 0.8 | 0.4 |
| 2nd | La CampaNahuelbuta | 1 | 0.9 | 1 | 1.8 |
| 2nd | La CampaNahuelbuta | 1 | 3 | 2.2 | 0.6 |
| 2nd | La CampaNahuelbuta | 1 | 0.5 | 0.5 | 0.3 |
| 2nd | La CampaNahuelbuta | 2 | 3.2 | 2.4 | 1.3 |
| 2nd | La CampaNahuelbuta | 2 | 1.4 | 1 | 0.9 |
| 2nd | La CampaNahuelbuta | 2 | 2.4 | 3.5 | 0.5 |
| 2nd | La CampaNahuelbuta | 2 | 2.2 | 3.5 | 1.9 |
| 2nd | La CampaNahuelbuta | 2 | 2.5 | 4 | 3.4 |
| 2nd | La CampaNahuelbuta | 2 | 1 | 0.4 | 0.3 |
| 2nd | La CampaNahuelbuta | 2 | 0.8 | 1.7 | 0.4 |
| 2nd | La CampaNahuelbuta | 2 | 12.4 | 9.5 | 6 |
| 2nd | La CampaNahuelbuta | 3 | 1 | 0.8 | 0.7 |
| 2nd | La CampaNahuelbuta | 3 | 1 | 0.9 | 0.6 |
| 2nd | La CampaNahuelbuta | 3 | 1 | 1.1 | 1.2 |
| 2nd | La CampaNahuelbuta | 3 | 1.3 | 1.4 | 1.8 |
| 2nd | La CampaNahuelbuta | 3 | 0.4 | 0.7 | 0.4 |
| 2nd | La CampaNahuelbuta | 3 | 2.9 | 3.3 | 2.3 |
| 2nd | La CampaNahuelbuta | 3 | 6.5 | 7.4 | 3.1 |
| 2nd | La CampaNahuelbuta | 3 | 1.2 | 1 | 3.2 |
| 2nd | La CampaNahuelbuta | 3 | 1.1 | 1.1 | 0.8 |
| 2nd | La CampaNahuelbuta | 3 | 0.9 | 0.8 | 0.6 |
| 2nd | La CampaNahuelbuta | 4 | 0.6 | 0.7 | 0.2 |
| 2nd | La CampaNahuelbuta | 4 | 0.5 | 0.5 | 0.3 |
| 2nd | La CampaNahuelbuta | 4 | 1 | 1 | 0.7 |
| 2nd | La CampaNahuelbuta | 4 | 0.7 | 0.7 | 0.3 |
| 2nd | La CampaNahuelbuta | 4 | 0.5 | 0.6 | 0.4 |
| 2nd | La CampaNahuelbuta | 4 | 0.4 | 0.4 | 0.3 |
| 2nd | La CampaNahuelbuta | 4 | 0.5 | 0.4 | 0.3 |
| 2nd | La CampaNahuelbuta | 4 | 0.8 | 0.6 | 0.5 |
| 2nd | La CampaNahuelbuta | 4 | 1 | 0.9 | 0.4 |
| 2nd | La CampaNahuelbuta | 4 | 0.5 | 0.4 | 0.2 |
| 2nd | La CampaNahuelbuta | 4 | 0.5 | 0.5 | 0.3 |
| 2nd | La CampaNahuelbuta | 5 | 1.2 | 1.3 | 0.9 |
| 2nd | La CampaNahuelbuta | 5 | 0.6 | 1 | 0.3 |
| 2nd | La CampaNahuelbuta | 5 | 0.9 | 0.4 | 0.3 |
| 2nd | La CampaNahuelbuta | 5 | 0.5 | 0.4 | 0.3 |
| 2nd | La CampaNahuelbuta | 5 | 0.4 | 0.3 | 0.4 |
| 2nd | La CampaNahuelbuta | 5 | 0.5 | 0.4 | 0.4 |
| 2nd | La CampaNahuelbuta | 5 | 0.6 | 0.7 | 0.5 |
| 2nd | La CampaNahuelbuta | 6 | 0.6 | 0.6 | 0.4 |
| 2nd | La CampaNahuelbuta | 6 | 0.7 | 0.4 | 0.3 |
| 2nd | La CampaNahuelbuta | 6 | 0.8 | 0.7 | 0.7 |
| 2nd | La CampaNahuelbuta | 7 | 1.4 | 2.1 | 0.9 |
| 2nd | La CampaNahuelbuta | 7 | 1 | 1.5 | 1.3 |
| 2nd | La CampaNahuelbuta | 7 | 0.8 | 1.1 | 0.3 |
| 2nd | La CampaNahuelbuta | 7 | 0.6 | 0.6 | 0.5 |
| 2nd | La CampaNahuelbuta | 7 | 10 | 6.5 | 9 |
| 2nd | La CampaNahuelbuta | 7 | 0.6 | 0.6 | 0.5 |
| 2nd | La CampaNahuelbuta | 7 | 0.7 | 0.6 | 0.3 |
| 2nd | La CampaNahuelbuta | 7 | 0.8 | 0.9 | 0.3 |
| 2nd | La CampaNahuelbuta | 7 | 0.7 | 1.7 | 1.9 |
| 2nd | La CampaNahuelbuta | 7 | 3.2 | 4.6 | 4.1 |
| 2nd | La CampaNahuelbuta | 7 | 0.9 | 1 | 0.7 |
| 2nd | La CampaNahuelbuta | 7 | 0.5 | 0.6 | 0.6 |
| 2nd | La CampaNahuelbuta | 7 | 0.6 | 0.8 | 1 |
| 2nd | La CampaNahuelbuta | 7 | 0.7 | 0.6 | 0.6 |
| 2nd | La CampaNahuelbuta | 8 | 0.5 | 0.5 | 0.8 |
| 2nd | La CampaNahuelbuta | 8 | 0.4 | 0.5 | 0.5 |
| 2nd | La CampaNahuelbuta | 9 | 0.6 | 0.5 | 0.6 |
| 2nd | La CampaNahuelbuta | 9 | 0.7 | 2.2 | 1.1 |
| 2nd | La CampaNahuelbuta | 9 | 1 | 1.8 | 1.4 |
| 2nd | La CampaNahuelbuta | 9 | 0.9 | 0.7 | 0.5 |
| 2nd | La CampaNahuelbuta | 9 | 1.6 | 1.9 | 1 |
| 2nd | La CampaNahuelbuta | 9 | 0.5 | 0.4 | 0.4 |
| 2nd | La CampaNahuelbuta | 9 | 0.6 | 0.7 | 0.3 |
| 2nd | La CampaNahuelbuta | 9 | 0.6 | 0.5 | 0.7 |
| 2nd | La CampaNahuelbuta | 9 | 5.2 | 6.9 | 3.4 |
| 2nd | La CampaNahuelbuta | 9 | 0.5 | 0.6 | 0.4 |
| 2nd | La CampaNahuelbuta | 9 | 0.5 | 0.4 | 0.4 |
| 2nd | La CampaNahuelbuta | 9 | 0.3 | 0.3 | 0.2 |
| 2nd | La CampaNahuelbuta | 9 | 0.5 | 0.4 | 0.2 |
| 2nd | La CampaNahuelbuta | 9 | 0.8 | 1.1 | 1.7 |
| 2nd | La CampaNahuelbuta | 9 | 0.4 | 0.3 | 0.2 |
| 2nd | La CampaNahuelbuta | 10 | 10 | 12 | 7 |
| 2nd | La CampaNahuelbuta | 10 | 4.7 | 9 | 3.6 |
| 2nd | La CampaNahuelbuta | 10 | 3 | 7 | 2.1 |
| 2nd | La CampaNahuelbuta | 10 | 1.5 | 1.7 | 1.1 |
| 2nd | La CampaNahuelbuta | 10 | 3 | 2.7 | 1.2 |
| 2nd | La CampaNahuelbuta | 10 | 5 | 3.5 | 5 |
| 2nd | La CampaNahuelbuta | 10 | 4.5 | 3.6 | 1.9 |
| 2nd | La CampaNahuelbuta | 10 | 6.5 | 5.7 | 9 |
| 2nd | La CampaNahuelbuta | 10 | 4.5 | 5.3 | 2.8 |
| 2nd | La CampaNahuelbuta | 10 | 9 | 9.1 | 12.5 |
| 2nd | La CampaNahuelbuta | 10 | 5 | 6.7 | 3.2 |
| 2nd | La CampaNahuelbuta | 10 | 7 | 5 | 11 |
| 2nd | La CampaNahuelbuta | 10 | 1.5 | 3.6 | 0.6 |
| 2nd | La CampaNahuelbuta | 10 | 3 | 2 | 1.7 |
| 2nd | La CampaNahuelbuta | 10 | 3.3 | 0.7 | 0.9 |
| 2nd | La CampaNahuelbuta | 10 | 4.5 | 2.5 | 3 |
| 2nd | La CampaNahuelbuta | 10 | 5.6 | 5.7 | 4 |
| 2nd | La CampaNahuelbuta | 10 | 5 | 7 | 7.6 |
| 2nd | La CampaNahuelbuta | 10 | 4.5 | 5 | 9.7 |
| 2nd | La CampaNahuelbuta | 10 | 5.5 | 4 | 8.7 |
| 2nd | La CampaNahuelbuta | 10 | 4 | 4.6 | 6 |
| 2nd | La CampaNahuelbuta | 10 | 5 | 3 | 3 |
| 2nd | La CampaNahuelbuta | 10 | 5 | 3.9 | 3 |
| 2nd | La CampaNahuelbuta | 10 | 3 | 7 | 2.4 |
| 2nd | La CampaNahuelbuta | 10 | 5 | 7.9 | 7.6 |
| 2nd | La CampaNahuelbuta | 10 | 5 | 8 | 6.2 |
| 2nd | La CampaNahuelbuta | 10 | 5 | 4.9 | 1.9 |
| 2nd | La CampaNahuelbuta | 10 | 3.5 | 6 | 1.9 |
| 2nd | La CampaNahuelbuta | 10 | 4.9 | 4.5 | 7 |
| 2nd | La CampaNahuelbuta | 10 | 2.9 | 4.2 | 6 |
| 2nd | La CampaNahuelbuta | 10 | 5.5 | 4.7 | 2 |
| 2nd | La CampaNahuelbuta | 10 | 11 | 9 | 7.1 |
| 2nd | La CampaNahuelbuta | 10 | 9.8 | 9 | 7.1 |
| 2nd | La CampaNahuelbuta | 10 | 4 | 6 | 3.1 |
| 2nd | La CampaNahuelbuta | 11 | 6 | 4.5 | 2.5 |
| 2nd | La CampaNahuelbuta | 11 | 3.5 | 4.5 | 3.5 |
| 2nd | La CampaNahuelbuta | 11 | 5.5 | 4.5 | 3.9 |
| 2nd | La CampaNahuelbuta | 11 | 3 | 5 | 3.5 |
| 2nd | La CampaNahuelbuta | 11 | 5.5 | 6 | 5.2 |
| 2nd | La CampaNahuelbuta | 11 | 4.3 | 4.5 | 3.9 |
| 2nd | La CampaNahuelbuta | 11 | 2 | 4 | 2 |
| 2nd | La CampaNahuelbuta | 11 | 3.9 | 3.2 | 1.8 |
| 2nd | La CampaNahuelbuta | 11 | 3 | 1.9 | 1.4 |
| 2nd | La CampaNahuelbuta | 11 | 3.5 | 4 | 2.4 |
| 2nd | La CampaNahuelbuta | 11 | 8 | 11.5 | 6.9 |
| 2nd | La CampaNahuelbuta | 11 | 3 | 4 | 4.1 |
| 2nd | La CampaNahuelbuta | 11 | 3.5 | 2 | 1.4 |
| 2nd | La CampaNahuelbuta | 11 | 6 | 8.5 | 3.2 |
| 2nd | La CampaNahuelbuta | 11 | 12 | 10.5 | 3.4 |
| 2nd | La CampaNahuelbuta | 11 | 2 | 4.1 | 2.8 |
| 2nd | La CampaNahuelbuta | 11 | 3.4 | 3.8 | 1.7 |
| 2nd | La CampaNahuelbuta | 11 | 28 | 29 | 35.5 |
| 2nd | La CampaNahuelbuta | 11 | 4 | 3.9 | 2.8 |
| 2nd | La CampaNahuelbuta | 11 | 3.5 | 3 | 2.1 |
| 2nd | La CampaNahuelbuta | 11 | 6 | 5 | 2.7 |
| 2nd | La CampaNahuelbuta | 11 | 7.5 | 4 | 2.4 |
| 2nd | La CampaNahuelbuta | 11 | 3.6 | 9.6 | 5.3 |
| 2nd | La CampaNahuelbuta | 11 | 2.9 | 2.4 | 1.5 |
| 2nd | La CampaNahuelbuta | 11 | 5 | 6.7 | 6.2 |
| 2nd | La CampaNahuelbuta | 12 | 0.9 | 1.7 | 0.6 |
| 2nd | La CampaNahuelbuta | 12 | 9 | 6.5 | 6.6 |
| 2nd | La CampaNahuelbuta | 12 | 2.4 | 5 | 2.5 |
| 2nd | La CampaNahuelbuta | 12 | 0.8 | 0.8 | 0.6 |
| 2nd | La CampaNahuelbuta | 12 | 6.4 | 6.7 | 9.1 |
| 2nd | La CampaNahuelbuta | 12 | 1.7 | 1.5 | 1.5 |
| 2nd | La CampaNahuelbuta | 12 | 5 | 5.6 | 3.2 |
| 2nd | La CampaNahuelbuta | 12 | 4 | 3.7 | 6.1 |
| 2nd | La CampaNahuelbuta | 12 | 6 | 3.7 | 5.1 |
| 2nd | La CampaNahuelbuta | 12 | 9.1 | 6.8 | 9.9 |
| 2nd | La CampaNahuelbuta | 12 | 3 | 3.6 | 2.2 |
| 2nd | La CampaNahuelbuta | 13 | 0.5 | 0.5 | 0.4 |
| 2nd | La CampaNahuelbuta | 13 | 0.8 | 0.8 | 1.9 |
| 2nd | La CampaNahuelbuta | 13 | 0.6 | 0.7 | 0.4 |
| 2nd | La CampaNahuelbuta | 13 | 0.6 | 0.6 | 0.3 |
| 2nd | La CampaNahuelbuta | 13 | 0.6 | 0.5 | 0.4 |
| 2nd | La CampaNahuelbuta | 13 | 0.7 | 0.5 | 0.5 |
| 2nd | La CampaNahuelbuta | 13 | 0.3 | 0.8 | 0.6 |
| 2nd | La CampaNahuelbuta | 13 | 0.8 | 0.6 | 0.4 |
| 2nd | La CampaNahuelbuta | 13 | 1 | 0.9 | 0.7 |
| 2nd | La CampaNahuelbuta | 13 | 0.7 | 0.6 | 0.4 |
| 2nd | La CampaNahuelbuta | 13 | 0.6 | 0.8 | 0.4 |
| 2nd | La CampaNahuelbuta | 13 | 0.5 | 0.5 | 0.3 |
| 2nd | La CampaNahuelbuta | 13 | 1 | 1.2 | 1.1 |
| 2nd | La CampaNahuelbuta | 13 | 0.6 | 1.2 | 0.4 |
| 2nd | La CampaNahuelbuta | 13 | 0.8 | 0.8 | 0.5 |
| 2nd | La CampaNahuelbuta | 14 | 1.4 | 2.4 | 0.9 |
| 2nd | La CampaNahuelbuta | 14 | 1.3 | 1 | 2.8 |
| 2nd | La CampaNahuelbuta | 14 | 2.7 | 2.1 | 1.8 |
| 2nd | La CampaNahuelbuta | 14 | 3.2 | 3.9 | 2.8 |
| 2nd | La CampaNahuelbuta | 14 | 3.4 | 3.2 | 2.6 |
| 2nd | La CampaNahuelbuta | 14 | 4 | 2.9 | 1.7 |
| 2nd | La CampaNahuelbuta | 14 | 9.3 | 3.9 | 2.6 |
| 2nd | La CampaNahuelbuta | 14 | 6.4 | 11 | 4.5 |
| 2nd | La CampaNahuelbuta | 14 | 1.5 | 1.3 | 1.1 |
| 2nd | La CampaNahuelbuta | 14 | 4.3 | 5 | 4.1 |
| 2nd | La CampaNahuelbuta | 15 | 1.4 | 3 | 0.5 |
| 2nd | La CampaNahuelbuta | 15 | 1 | 2.4 | 0.3 |
| 2nd | La CampaNahuelbuta | 15 | 0.8 | 0.9 | 0.4 |
| 2nd | La CampaNahuelbuta | 15 | 0.6 | 0.9 | 0.6 |
| 2nd | La CampaNahuelbuta | 15 | 0.4 | 0.6 | 0.3 |
| 2nd | La CampaNahuelbuta | 15 | 1.4 | 2.6 | 1.1 |
| 2nd | La CampaNahuelbuta | 15 | 1.5 | 2 | 0.7 |
| 2nd | La CampaNahuelbuta | 15 | 0.9 | 0.9 | 0.5 |
| 2nd | La CampaNahuelbuta | 15 | 1 | 0.7 | 0.6 |
| 2nd | La CampaNahuelbuta | 15 | 1.2 | 1.3 | 0.6 |
| 2nd | La CampaNahuelbuta | 15 | 1 | 1 | 0.9 |
| 2nd | La CampaNahuelbuta | 15 | 1.4 | 1.6 | 0.9 |
| 2nd | La CampaNahuelbuta | 15 | 1.2 | 1.3 | 0.9 |
| 2nd | La CampaNahuelbuta | 15 | 0.8 | 1.7 | 1 |
| 2nd | La CampaNahuelbuta | 15 | 3.5 | 3 | 19 |
| 2nd | La CampaNahuelbuta | 15 | 0.8 | 0.8 | 0.7 |
| 2nd | La CampaNahuelbuta | 16 | 0.8 | 0.6 | 0.4 |
| 2nd | La CampaNahuelbuta | 16 | 1 | 0.8 | 0.3 |
| 2nd | La CampaNahuelbuta | 16 | 0.9 | 0.5 | 0.5 |
| 2nd | La CampaNahuelbuta | 16 | 1.6 | 0.9 | 1.1 |
| 2nd | La CampaNahuelbuta | 16 | 0.5 | 0.7 | 0.5 |
| 2nd | La CampaNahuelbuta | 16 | 0.7 | 0.5 | 0.5 |
| 2nd | La CampaNahuelbuta | 16 | 1.9 | 2 | 1.3 |
| 2nd | La CampaNahuelbuta | 16 | 1 | 0.8 | 0.7 |
| 2nd | La CampaNahuelbuta | 17 | 0.7 | 0.6 | 0.6 |
| 2nd | La CampaNahuelbuta | 17 | 0.3 | 0.4 | 0.3 |
| 2nd | La CampaNahuelbuta | 17 | 0.8 | 1 | 0.3 |
| 2nd | La CampaNahuelbuta | 17 | 1.3 | 1.4 | 1 |
| 2nd | La CampaNahuelbuta | 17 | 0.6 | 0.5 | 0.8 |
| 2nd | La CampaNahuelbuta | 17 | 0.4 | 0.3 | 0.3 |
| 2nd | La CampaNahuelbuta | 17 | 0.5 | 0.6 | 0.6 |
| 2nd | La CampaNahuelbuta | 17 | 0.6 | 1 | 1.1 |
| 2nd | La CampaNahuelbuta | 17 | 0.8 | 1.2 | 1 |
| 2nd | La CampaNahuelbuta | 17 | 1.9 | 1.2 | 1 |
| 2nd | La CampaNahuelbuta | 17 | 2.1 | 1.8 | 2.9 |
| 2nd | La CampaNahuelbuta | 17 | 0.7 | 0.5 | 0.4 |
| 2nd | La CampaNahuelbuta | 17 | 1.5 | 2 | 1.2 |
| 2nd | La CampaNahuelbuta | 17 | 0.5 | 0.7 | 0.3 |
| 2nd | La CampaNahuelbuta | 17 | 1.4 | 3.5 | 2.2 |
| 2nd | La CampaNahuelbuta | 17 | 1.3 | 0.9 | 0.6 |
| 2nd | La CampaNahuelbuta | 17 | 1.3 | 2.4 | 0.7 |
| 2nd | La CampaNahuelbuta | 18 | 4.3 | 5.5 | 9.3 |
| 2nd | La CampaNahuelbuta | 18 | 1.4 | 5 | 0.5 |
| 2nd | La CampaNahuelbuta | 18 | 4 | 6 | 2.1 |
| 2nd | La CampaNahuelbuta | 18 | 6 | 7 | 6 |
| 2nd | La CampaNahuelbuta | 18 | 1.2 | 1.5 | 0.3 |
| 2nd | La CampaNahuelbuta | 18 | 8 | 9.3 | 5 |
| 2nd | La CampaNahuelbuta | 18 | 4 | 7.3 | 2.5 |
| 2nd | La CampaNahuelbuta | 18 | 2 | 2.4 | 1.8 |
| 2nd | La CampaNahuelbuta | 18 | 5 | 6.4 | 2.9 |
| 2nd | La CampaNahuelbuta | 18 | 5 | 7 | 6.3 |
| 2nd | La CampaNahuelbuta | 19 | 3.7 | 5 | 5.4 |
| 2nd | La CampaNahuelbuta | 19 | 3 | 2.6 | 1.9 |
| 2nd | La CampaNahuelbuta | 19 | 1.2 | 1.7 | 1.6 |
| 2nd | La CampaNahuelbuta | 19 | 5 | 4.4 | 3.8 |
| 2nd | La CampaNahuelbuta | 19 | 3.6 | 4 | 0.8 |
| 2nd | La CampaNahuelbuta | 19 | 2.2 | 0.3 | 3.6 |
| 2nd | La CampaNahuelbuta | 19 | 0.6 | 1 | 0.4 |
| 2nd | La CampaNahuelbuta | 19 | 3 | 3.5 | 4.3 |
| 2nd | La CampaNahuelbuta | 19 | 1 | 2 | 1.5 |
| 2nd | La CampaNahuelbuta | 19 | 3 | 3.4 | 2.1 |
| 2nd | La CampaNahuelbuta | 19 | 1 | 1.3 | 1.9 |
| 2nd | La CampaNahuelbuta | 19 | 0.7 | 0.8 | 0.8 |
| 2nd | La CampaNahuelbuta | 20 | 2.5 | 4 | 3.5 |
| 2nd | La CampaNahuelbuta | 20 | 1 | 1.5 | 1.6 |
| 2nd | La CampaNahuelbuta | 20 | 1.2 | 2.7 | 0.6 |
| 2nd | La CampaNahuelbuta | 20 | 4 | 5.5 | 11.2 |
| 2nd | La CampaNahuelbuta | 20 | 4 | 5.5 | 11.2 |
| 2nd | La CampaNahuelbuta | 20 | 6 | 5.8 | 7 |
| 2nd | La CampaNahuelbuta | 20 | 10 | 6 | 4.8 |
| 2nd | La CampaNahuelbuta | 20 | 0.4 | 0.4 | 0.8 |
| 2nd | La CampaNahuelbuta | 20 | 0.5 | 0.4 | 0.3 |
| 2nd | La CampaNahuelbuta | 20 | 1 | 1.2 | 0.5 |
| 2nd | La CampaNahuelbuta | 20 | 8.6 | 7.8 | 6 |
| 2nd | La CampaNahuelbuta | 20 | 2.7 | 5.9 | 4.1 |
| 2nd | La CampaNahuelbuta | 20 | 0.9 | 1 | 0.7 |
| 2nd | La CampaNahuelbuta | 20 | 3.5 | 5.9 | 4.6 |
| 2nd | La CampaNahuelbuta | 20 | 5.8 | 6.5 | 4.6 |
| 2nd | Nahuelbuta | 1 | 2.9 | 3.4 | 1.7 |
| 2nd | Nahuelbuta | 1 | 7 | 9.1 | 3.1 |
| 2nd | Nahuelbuta | 1 | 0.7 | 2 | 0.4 |
| 2nd | Nahuelbuta | 1 | 3 | 3.1 | 1 |
| 2nd | Nahuelbuta | 1 | 12 | 11.7 | 7.3 |
| 2nd | Nahuelbuta | 1 | 4.1 | 5.7 | 3 |
| 2nd | Nahuelbuta | 1 | 3.5 | 6 | 2.8 |
| 2nd | Nahuelbuta | 1 | 2.3 | 1.7 | 1.4 |
| 2nd | Nahuelbuta | 2 | 0.8 | 0.6 | 0.9 |
| 2nd | Nahuelbuta | 2 | 2 | 3 | 1.8 |
| 2nd | Nahuelbuta | 2 | 1 | 2.5 | 0.9 |
| 2nd | Nahuelbuta | 2 | 2 | 1.5 | 0.5 |
| 2nd | Nahuelbuta | 2 | 1.5 | 1.2 | 1.9 |
| 2nd | Nahuelbuta | 2 | 0.8 | 1.1 | 0.5 |
| 2nd | Nahuelbuta | 2 | 0.5 | 0.4 | 1.2 |
| 2nd | Nahuelbuta | 2 | 0.4 | 0.3 | 1.7 |
| 2nd | Nahuelbuta | 2 | 1 | 0.9 | 0.8 |
| 2nd | Nahuelbuta | 3 | 3 | 3.4 | 2.8 |
| 2nd | Nahuelbuta | 3 | 5 | 5.5 | 6.1 |
| 2nd | Nahuelbuta | 3 | 7 | 4 | 2.8 |
| 2nd | Nahuelbuta | 3 | 12 | 10.4 | 7.5 |
| 2nd | Nahuelbuta | 3 | 2 | 3.4 | 3.2 |
| 2nd | Nahuelbuta | 4 | 0.6 | 0.7 | 0.3 |
| 2nd | Nahuelbuta | 4 | 0.4 | 0.4 | 0.7 |
| 2nd | Nahuelbuta | 4 | 0.5 | 0.8 | 1 |
| 2nd | Nahuelbuta | 4 | 1 | 1 | 0.8 |
| 2nd | Nahuelbuta | 4 | 4.5 | 6.8 | 9 |
| 2nd | Nahuelbuta | 4 | 1.2 | 1 | 1.5 |
| 2nd | Nahuelbuta | 4 | 0.5 | 0.4 | 0.6 |
| 2nd | Nahuelbuta | 4 | 0.4 | 0.4 | 0.3 |
| 2nd | Nahuelbuta | 4 | 18 | 15 | 8.5 |
| 2nd | Nahuelbuta | 4 | 0.5 | 0.6 | 0.3 |
| 2nd | Nahuelbuta | 4 | 1.5 | 1.3 | 1 |
| 2nd | Nahuelbuta | 4 | 1 | 1.8 | 1.7 |
| 2nd | Nahuelbuta | 5 | 3 | 2.6 | 1.4 |
| 2nd | Nahuelbuta | 5 | 2.3 | 2.5 | 1.6 |
| 2nd | Nahuelbuta | 5 | 9 | 9 | 3.8 |
| 2nd | Nahuelbuta | 5 | 0.8 | 1.2 | 0.6 |
| 2nd | Nahuelbuta | 5 | 0.4 | 0.4 | 0.5 |
| 2nd | Nahuelbuta | 5 | 1 | 0.8 | 1.2 |
| 2nd | Nahuelbuta | 5 | 1.4 | 1 | 3.7 |
| 2nd | Nahuelbuta | 5 | 0.5 | 0.7 | 0.6 |
| 2nd | Nahuelbuta | 6 | 1.5 | 1.7 | 1.4 |
| 2nd | Nahuelbuta | 6 | 4 | 4.6 | 2.7 |
| 2nd | Nahuelbuta | 6 | 0.9 | 0.7 | 0.6 |
| 2nd | Nahuelbuta | 6 | 2.5 | 3.8 | 1.8 |
| 2nd | Nahuelbuta | 6 | 1.4 | 1.3 | 0.6 |
| 2nd | Nahuelbuta | 7 | 2 | 2.4 | 1.8 |
| 2nd | Nahuelbuta | 7 | 3 | 3.8 | 0.5 |
| 2nd | Nahuelbuta | 8 | 1.9 | 3.4 | 4.7 |
| 2nd | Nahuelbuta | 8 | 3 | 5.5 | 3.6 |
| 2nd | Nahuelbuta | 8 | 2 | 2.6 | 1.6 |
| 2nd | Nahuelbuta | 9 | NA | NA | NA |
| 2nd | Nahuelbuta | 10 | NA | NA | NA |
| 2nd | Nahuelbuta | 11 | NA | NA | NA |
| 2nd | Nahuelbuta | 12 | NA | NA | NA |
| 2nd | Nahuelbuta | 13 | 1.7 | 1.8 | 1.5 |
| 2nd | Nahuelbuta | 13 | 3 | 2.5 | 1.5 |
| 2nd | Nahuelbuta | 13 | 2 | 1.8 | 0.4 |
| 2nd | Nahuelbuta | 13 | 3 | 4 | 1.6 |
| 2nd | Nahuelbuta | 13 | 1.4 | 1.9 | 1.4 |
| 2nd | Nahuelbuta | 13 | 5 | 11 | 4.7 |
| 2nd | Nahuelbuta | 13 | 5 | 9 | 7.9 |
| 2nd | Nahuelbuta | 13 | 4 | 8 | 4.2 |
| 2nd | Nahuelbuta | 13 | 1.5 | 2.5 | 2.4 |
| 2nd | Nahuelbuta | 14 | 2 | 2 | 2.8 |
| 2nd | Nahuelbuta | 14 | 4 | 5 | 2.4 |
| 2nd | Nahuelbuta | 14 | 0.6 | 1.3 | 0.5 |
| 2nd | Nahuelbuta | 14 | 2.7 | 2.9 | 0.8 |
| 2nd | Nahuelbuta | 14 | 2 | 1.8 | 1 |
| 2nd | Nahuelbuta | 14 | 2 | 1.9 | 2.2 |
| 2nd | Nahuelbuta | 14 | 4.3 | 3.9 | 1.8 |
| 2nd | Nahuelbuta | 14 | 1.4 | 1.8 | 0.7 |
| 2nd | Nahuelbuta | 14 | 2 | 1.8 | 1 |
| 2nd | Nahuelbuta | 14 | 1.6 | 1.7 | 1.8 |
| 2nd | Nahuelbuta | 14 | 1.5 | 2 | 0.9 |
| 2nd | Nahuelbuta | 15 | 1 | 0.8 | 0.5 |
| 2nd | Nahuelbuta | 15 | 3 | 2.9 | 2.4 |
| 2nd | Nahuelbuta | 15 | 1.5 | 1.4 | 1.2 |
| 2nd | Nahuelbuta | 15 | 2.1 | 2 | 1.4 |
| 2nd | Nahuelbuta | 15 | 3 | 2.3 | 3 |
| 2nd | Nahuelbuta | 15 | 1.4 | 4.6 | 5.5 |
| 2nd | Nahuelbuta | 15 | 6.9 | 7.1 | 4.7 |
| 2nd | Nahuelbuta | 16 | 1 | 0.7 | 0.5 |
| 2nd | Nahuelbuta | 16 | 0.4 | 0.5 | 0.7 |
| 2nd | Nahuelbuta | 16 | 6 | 11.5 | 4.8 |
| 2nd | Nahuelbuta | 16 | 11 | 12 | 6 |
| 2nd | Nahuelbuta | 16 | 5 | 3.5 | 2.7 |
| 2nd | Nahuelbuta | 16 | 9 | 10 | 6.4 |
| 2nd | Nahuelbuta | 16 | 1 | 1.3 | 0.5 |
| 2nd | Nahuelbuta | 16 | 0.8 | 0.8 | 0.3 |
| 2nd | Nahuelbuta | 16 | 3 | 3.8 | 2.4 |
| 2nd | Nahuelbuta | 17 | 1 | 1.4 | 0.8 |
| 2nd | Nahuelbuta | 17 | 1 | 1.3 | 1.5 |
| 2nd | Nahuelbuta | 17 | 1.5 | 0.8 | 0.6 |
| 2nd | Nahuelbuta | 17 | 1.3 | 1.5 | 0.5 |
| 2nd | Nahuelbuta | 17 | 1 | 1.2 | 0.8 |
| 2nd | Nahuelbuta | 17 | 2.5 | 2.6 | 2.1 |
| 2nd | Nahuelbuta | 17 | 2 | 1.5 | 2.2 |
| 2nd | Nahuelbuta | 18 | 2 | 1.4 | 0.9 |
| 2nd | Nahuelbuta | 18 | 1 | 1 | 1.5 |
| 2nd | Nahuelbuta | 18 | 1 | 0.8 | 0.6 |
| 2nd | Nahuelbuta | 19 | NA | NA | NA |
| 2nd | Nahuelbuta | 20 | NA | NA | NA |
